# HIF1/2-exerted control over glycolytic gene expression is not functionally relevant for glycolysis in human leukemic stem/progenitor cells

**DOI:** 10.1186/s40170-019-0206-y

**Published:** 2019-12-27

**Authors:** Albertus T. J. Wierenga, Alan Cunningham, Ayşegül Erdem, Nuria Vilaplana Lopera, Annet Z. Brouwers-Vos, Maurien Pruis, André B. Mulder, Ulrich L. Günther, Joost H. A. Martens, Edo Vellenga, Jan Jacob Schuringa

**Affiliations:** 1 Department of Experimental Hematology, University Medical Center Groningen, University of Groningen, Hanzeplein 1, Groningen, 9700RB The Netherlands; 2 Department of Laboratory Medicine, University Medical Center Groningen, University of Groningen, Hanzeplein 1, 9700RB Groningen, The Netherlands; 3 Institute of Cancer and Genomic Sciences, University of Birmingham, Birmingham, UK; 4 Department of Molecular Biology, Radboud Institute for Molecular Life Sciences (RIMLS), Nijmegen, The Netherlands

**Keywords:** Hypoxia induced factors (HIFs), Human hematopoietic stem cells, Acute myeloid leukemia (AML), Glycolysis, Hypoxia, BCR-ABL

## Abstract

**Background:**

Hypoxia-inducible factors (HIF)1 and 2 are transcription factors that regulate the homeostatic response to low oxygen conditions. Since data related to the importance of HIF1 and 2 in hematopoietic stem and progenitors is conflicting, we investigated the chromatin binding profiles of HIF1 and HIF2 and linked that to transcriptional networks and the cellular metabolic state.

**Methods:**

Genome-wide ChIPseq and ChIP-PCR experiments were performed to identify HIF1 and HIF2 binding sites in human acute myeloid leukemia (AML) cells and healthy CD34^+^ hematopoietic stem/progenitor cells. Transcriptome studies were performed to identify gene expression changes induced by hypoxia or by overexpression of oxygen-insensitive HIF1 and HIF2 mutants. Metabolism studies were performed by 1D-NMR, and glucose consumption and lactate production levels were determined by spectrophotometric enzyme assays. CRISPR-CAS9-mediated HIF1, HIF2, and ARNT^−/−^ lines were generated to study the functional consequences upon loss of HIF signaling, in vitro and in vivo upon transplantation of knockout lines in xenograft mice.

**Results:**

Genome-wide ChIP-seq and transcriptome studies revealed that overlapping HIF1- and HIF2-controlled loci were highly enriched for various processes including metabolism, particularly glucose metabolism, but also for chromatin organization, cellular response to stress and G protein-coupled receptor signaling. ChIP-qPCR validation studies confirmed that glycolysis-related genes but not genes related to the TCA cycle or glutaminolysis were controlled by both HIF1 and HIF2 in leukemic cell lines and primary AMLs, while in healthy human CD34^+^ cells these loci were predominantly controlled by HIF1 and not HIF2. However, and in contrast to our initial hypotheses, CRISPR/Cas9-mediated knockout of HIF signaling did not affect growth, internal metabolite concentrations, glucose consumption or lactate production under hypoxia, not even in vivo upon transplantation of knockout cells into xenograft mice.

**Conclusion:**

These data indicate that, while HIFs exert control over glycolysis but not OxPHOS gene expression in human leukemic cells, this is not critically important for their metabolic state. In contrast, inhibition of BCR-ABL did impact on glucose consumption and lactate production regardless of the presence of HIFs. These data indicate that oncogene-mediated control over glycolysis can occur independently of hypoxic signaling modules.

## Background

Hematopoietic stem cells (HSCs) and their leukemic stem cell (LSC) counterparts reside within the bone marrow microenvironment where they are surrounded by a wide variety of other cell types that together constitute the stem cell niche [1, 2]. Osteoblasts, osteoclasts, adipocytes, vascular endothelial cells, and various other stromal components provide the necessary factors that control stem cell fate such as self-renewal, quiescence, dormancy, survival and differentiation. Additionally, the bone marrow microenvironment where HSCs reside is rather hypoxic [3, 4], with the lowest O_2_ concentrations of 1.3% found in peri-sinusoidal regions [5]. It is widely assumed that these conditions in the niche further contribute to the quiescence and metabolic state of HSCs [6–8], although the molecular mechanisms that are involved are only beginning to be unraveled.

Hypoxia-inducible factors HIF1α and HIF2α act as oxygen sensors that are degraded under normoxic conditions but at lower oxygen levels HIF proteins are stabilized and bind to their co-factor ARNT (HIF1β), before translocating to the nucleus to initiate gene transcription [9–11]. HIFs have been characterized as important factors controlling cellular metabolism and self-renewal of HSCs [8, 12–15]. Hyperactivation of HIFs has been reported in many cancers [16, 17], including in leukemias where they might participate in the transformation process [18–21]. In chronic lymphocytic leukemia, metabolic plasticity in response to hypoxia has been described, where the rate of glucose consumption and lactate production was most affected [22]. This metabolic adaptation was shown to be HIF1-dependent and no longer possible when HIF1 was inhibited using chetomin [22]. In contrast, it has also been reported that HIF1 is dispensable for adult HSCs and that they do not require intrinsic HIF1 to be able to respond to injury [23] and the same was shown for HIF2 [24]. More in line with that latter notion, HIF1 has also been identified as a tumor suppressor whereby HIF1 loss resulted in enhanced leukemogenesis [25, 26]. These conflicting data indicate that the exact role of HIF1 in the hematopoietic system in health and disease remains far from clear, and also the role of HIF2 is still under debate. Despite a high homology between HIF1 and HIF2 suggesting a strong overlap in functionalities, specific cellular roles for HIF1 and HIF2 have been described as well. In part, this might also be dictated by their cell-type specific expression profiles, whereby HIF1 appears to be highest expressed in the most immature HSC compartment [12] while HIF2 might play a more prominent role in vascular endothelial cells [27]. One of the most well documented roles of HIFs has indeed been the upregulation of VEGF to induce angiogenesis [9, 28, 29]. Previously, we identified HIF2 as a downstream target of STAT5 and observed elevated glucose uptake in STAT5 activated HSCs [30]. Several genes associated with glucose metabolism were upregulated by STAT5 in an HIF2-dependent manner, including SLC2A1 and GYS2 [30].

Under hypoxia, it has been shown that HIF1 can regulate pyruvate dehydrogenase kinases (PDKs), thereby preventing entry of pyruvate into the tricarboxylic acid cycle (TCA), resulting in enhanced lactate production in quiescent HSCs [31]. Indeed, an increasing number of papers have indicated that to maintain a quiescent stem cell state, HSCs wire their metabolic state towards glycolysis. HSCs self-renewal is better maintained when mitochondrial activity is maintained low [32] which might in part rely on mitochondrial clearance via mitophagy [33]. In line with this, the reduction of reactive oxygen species (ROS) by antioxidants maintains stemness in serial transplantation experiments [34] and also in leukemia it was proposed that the most immature LSCs with engraftment potential reside within the ROS^low^ fraction [35]. Upon lineage commitment, the PTEN-like mitochondrial phosphatase PTPMT1 primes the switch to mitochondrial oxidative phosphorylation to support the energy demands in differentiating HSCs [36]. Together, these studies highlight that distinct metabolic programs exist in quiescent versus actively cycling normal HSCs [37–39], although it is currently not clear how these programs are controlled at the molecular level.

Although the role of HIFs in HSCs has remained controversial, they do present as clear potential candidates to control the metabolic state of cells. By performing transcriptome studies, we and others previously identified that metabolism-associated genes can be activated by HIFs [9, 10, 40]. In leukemias, we have observed that various oncogenes can impose hypoxic signaling on normal human hematopoietic stem and progenitor cells even when grown under normoxic conditions [41–43]. Here, we set out to identify the direct HIF1 and HIF2 targets at the chromatin level in the human hematopoietic system by performing genome-wide ChIP-seq analyses, coupled to transcriptome and metabolome changes induced by HIFs or hypoxia. We report that, while HIFs can exert control over glycolysis but not OxPHOS pathways in human leukemic cells, this is not critically important for their metabolic state.

## Methods

### Cell culture and lentiviral transductions

Neonatal cord blood (CB) was obtained from healthy full-term pregnancies from the Obstetrics departments of the University Medical Center and Martini Hospital in Groningen, The Netherlands, after informed consent. The protocol was approved by the Medical Ethical Committee of the UMCG. Donors are informed about procedures and studies performed with CB by an information sheet that is read and signed by the donor, in line with regulations of the Medical Ethical Committee of the UMCG. CB CD34^+^ cells were isolated by density gradient separation, followed by the use of a hematopoietic progenitor magnetic associated cell sorting kit from Miltenyi Biotech according to the manufacturer’s instructions. Lentiviral transductions were essentially performed as described elsewhere [2–4].

### Generation of CRISPR/Cas9 lines

HIF1α, HIF2α, and ARNT were functionally knocked out by CRISPR/Cas9, as described in detail in the Additional file 7: Supplementary methods.

### ChIP-seq and ChIP-q-PCR

K562 cells were transduced with the lentiviral GFP-fusion vectors encoding HIF1α and HIF2α. 1 × 10^6^ EGFP positive cells were sorted and subsequently fixed in 1% formaldehyde for 10 min, quenched with 0.1 M glycine and processed for ChIP. Detailed methods including the used primers for ChIP-qPCR are described in the Additional files. ChIP reactions were performed using the following antibodies: anti-GFP (ab290, Abcam), anti-HIF1α (NB100-134, R&D systems), HIF2α (NB100-122, R&D systems), and ARNT (NB100-110, R&D systems). ChIP-seq data is deposited at GEO under GSE123461. Additional Materials and Methods can be found in the Additional files.

## Results

### HIF1 and HIF2 control glycolysis-related genes in human leukemic cells

Chromatin immunoprecipitation (ChIP)-sequencing was performed in order to identify HIF1 and HIF2-bound loci in human leukemic cells. The oxygen insensitive HIF mutants HIF1α(P402A,P564A)-EGFP and HIF2α(P405A,P531A)-EGFP (described previously in [40, 44]) were expressed as EGFP-fusion proteins in K562 cells. The HIF proline residues become hydroxylated under normoxic conditions which leads to their degradation, which is prevented by mutating these residues into alanines [45]. Anti-EGFP ChIPs were performed as outlined previously [30, 46], followed by deep sequencing. 50–60% of all identified peaks were located close to transcription start sites (TSSs, − 5kb to + 1 kb, Additional file 1: Figure S1a, Additional file 8: Table S1). Given the relatively large number of HIF binding sites distant from TSSs, we also analyzed whether HIFs would control lncRNAs or bind to super enhancers (SEs). Indeed, 13 to 15% of HIF1 and HIF2 peaks, respectively, were found to be located close to start sites of lncRNAs (Additional file 1: Figure S1b), while no significant enrichment of HIF binding was detected close to SEs (data not shown).

**Fig. 1:**
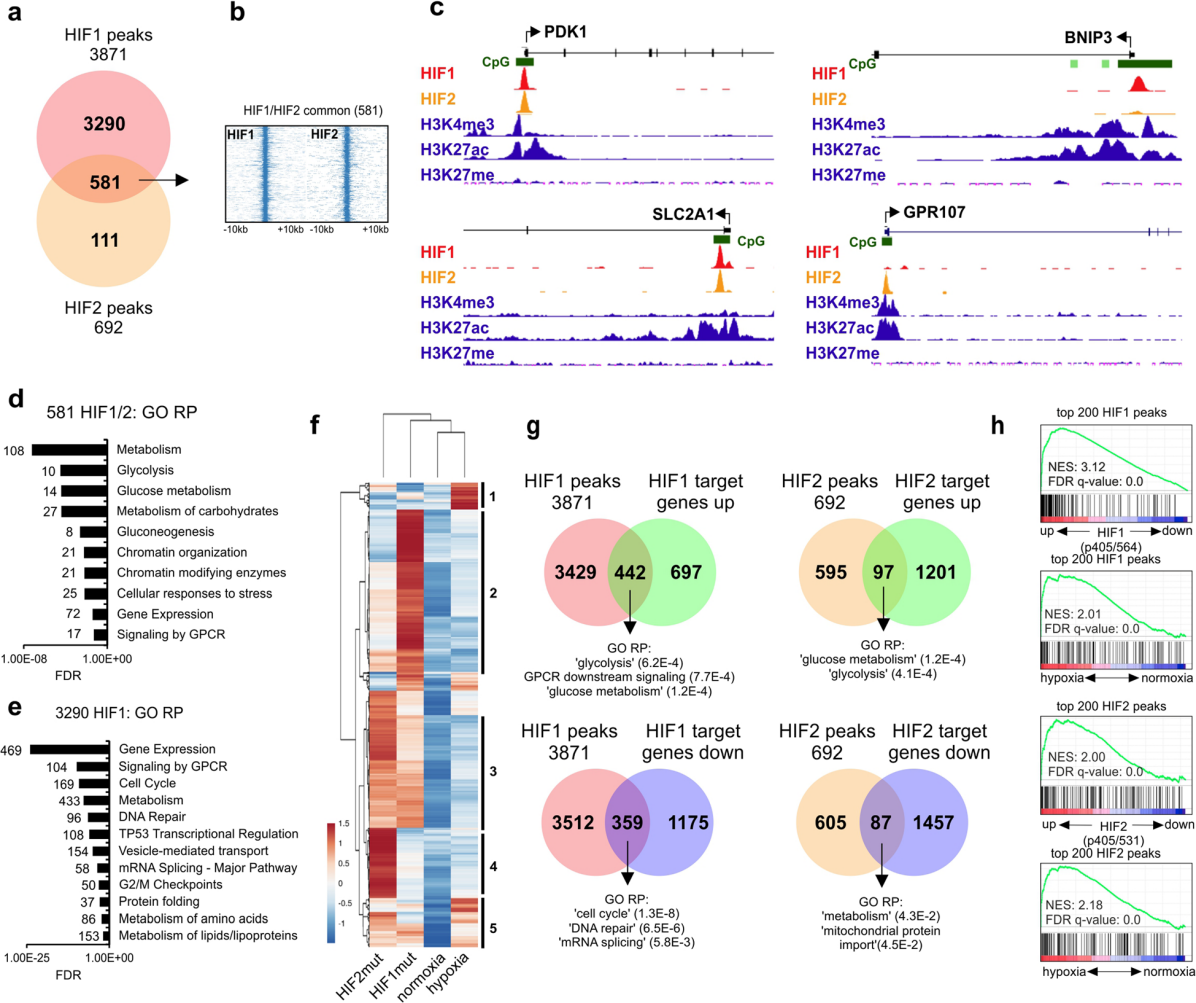
Identification of HIF1 and HIF2 chromatin binding sites in human leukemic cells. **a.** HIF1(P402A,P564A)-EGFP and HIF2(P405A,P531A)-EGFP fusions were expressed in K562 cells and anti-EGFP ChIPs were performed. VENN diagram depicts specific and overlapping peaks. H3K4me3, H3K27ac, and H3K27me3 K562 tracks were retrieved from Encode. **b.** Heatmaps of overlapping ChIP-seq peaks shown in **a**. **c** Left: representative examples of screenshots of loci bound by both HIF1 and HIF2, top right: representative screenshot of an HIF1-specific locus and bottom right: representative screenshot of an HIF2-specific locus. *y*-axis scales are set to 100 for HIF1 and HIF2, and to 50 for the other tracks. **d**–**e** GO analyses of gene loci bound by both HIF1/2 (**f**) or HIF1 only (**g**)**. f** Supervised clustering of genes upregulated (> 2-fold) under hypoxia or upon overexpression of HIF mutants in K562 cells. 1, genes predominantly upregulated by hypoxia; 2, genes predominantly upregulated by HIF1; 3, genes predominantly upregulated by HIF1/HIF2; 4, genes predominantly upregulated by HIF2; 5, genes upregulated by HIH1/HIF2 and hypoxia. **g** Overlap in HIF-bound loci determined by ChIP seq and HIF-induced gene expression changes. Reactome Pathway GO analyses was performed on overlapping genes as indicated. **h** GSEA analyses showing good correlations between HIF binding and HIF-induced gene expression, as well as between HIF binding and hypoxia-induced gene expression

Of the 3871 HIF1 peaks close to TSSs, 581 overlapped with HIF2 peaks (Fig. 1a–c). This overlapping set of HIF-bound loci was strongly enriched (FDR < 0.01) for various Reactome Pathway terms associated with metabolism, including “glycolysis,” “glucose metabolism,” and “gluconeogenesis,” but also for terms associated with chromatin organization and GPCR signaling (Fig. 1d, Additional file 1: Figure S1c). Loci more strongly bound by HIF1 were enriched for rather distinct processes such as cell cycle, DNA repair, vesicle-mediated transport and mRNA splicing (Fig. 1e), while no significant enrichment was observed for loci that were more strongly bound by HIF2 (data not shown).

A motif search on HIF1- and HIF2-bound promoters to identify other transcription factors that might act together with HIFs revealed that both HIF1 and HIF2 peaks were strongly enriched for NRF1, SP1, ELK1, and HIF motifs (within − 5 kb to + 1 kb around the TSS) (Additional file 8: Table S1). While no motifs were found to be specifically enriched around HIF2 peaks, a series of motifs were identified that were specifically present around HIF1 bound promoters, including binding sites for ATF3, CREB1, JUN, MAX, MYB, MYC, and ETS1 (Additional file 8: Table S1).

Chromatin binding was compared to gene expression changes (> 2-fold) induced by expression of the oxygen-insensitive HIF1α(P402A,P564A)-EGFP and HIF2α(P405A,P531A)-EGFP mutants in K562 cells, and also to transcriptome changes induced upon culturing of K562 cells under hypoxia (Fig. 1f). For the upregulated genes, a number of specific clusters could be identified that were predominantly upregulated by HIF1, HIF2, hypoxia, or all. Interestingly, this also allowed the identification of sets of genes controlled by hypoxia, independent of HIF transcription factors (Fig. 1f, cluster 1). Thirty-nine of the HIF1-upregulated genes (442/1139) were also directly bound by HIF1, while for the downregulated genes this was only 23% (359/1534) (Fig. 1g). For HIF2, the overlap between differentially expressed genes and those that were directly bound by HIF2 was smaller but for both HIF1 and HIF2 the overlapping bound and upregulated loci were strongly enriched for glycolysis-associated genes, while for HIF1 the overlapping downregulated loci were strongly enriched for reactome pathway terms “cell cycle,” “DNA repair,” and “mRNA splicing” (Fig. 1g, Additional file 9: Table S2). Furthermore, we ranked and identified the top 200 strongest bound loci by HIF1 and HIF2 and performed gene set enrichment analyses (GSEA) using differentially expressed gene sets induced by HIF1, HIF2 or hypoxia. These analyses again confirmed that the strongest bound genes are also the most strongly upregulated by HIF1 or HIF2, and also that these HIF-bound loci are strongly upregulated under hypoxic conditions (Fig. 1h).

To determine similarities and differences between HIF-bound loci across different cell types, we compared our ChIP-seq data from human leukemic cells with published data in breast cancer MCF7 cell lines [47, 48]. These analyses again confirmed that glucose metabolism and in particular glycolysis are processes that are controlled by both HIF1 and HIF2 independent of cell type (Additional file 1: Figure S1d, 1e).

Since HIF1 and HIF2 induced overlapping but also specific genes, we analyzed the co-occurrence of transcription factor (TF) binding motifs at HIF1 and HIF2-bound loci. Among the top scoring motifs, we observed NRF1 and ELK1 (Additional file 2: Figure S2a-c). Since for both of these, K562 ChIPseq tracks were available in ENCODE, we analyzed whether HIF1/2-bound loci would also be bound by NFR1 and ELK2. Indeed we observed that close to the TSS, binding of all factors was frequently observed, in particular in the case of glycolysis-related genes (Additional file 2: Figure S2d). While no HIF2-unique co-occurring TF motifs were found, we did find HIF1-unique co-occurring TF-binding motifs. Possibly, these differences also underlie HIF-specific target gene regulation but additional studies are required to obtain further insights into these phenomena.

**Fig. 2:**
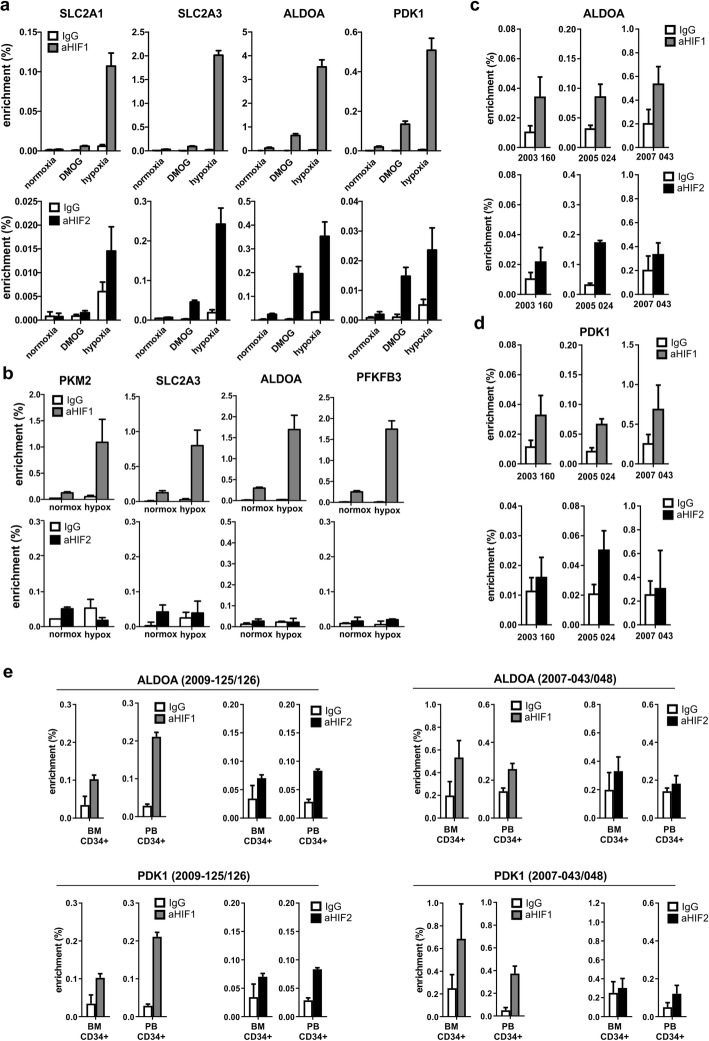
Validation of identified HIF1 and HIF2-bound loci in endogenous ChIP-PCRs. **a** HIF1 and HIF2 occupied loci identified by ChIPseq were validated in K562 using antibodies against endogenous HIF1 and HIF2. **b** HIF1 is more efficiently stabilized under hypoxia compared to HIF2 in CB CD34^+^ cells. Numbers below *x*-axis indicate patient sample numbers. **c–d** endogenous HIF1 and HIF2 ChIP PCRs on representative loci in primary AML CD34^+^ cells. **e** Endogenous HIF1 and HIF2 ChIP PCRs on representative loci in primary AML CD34^+^ cells derived from BM or PB. Numbers above graphs indicate patient sample numbers, whereby 2009-125 is derived from BM and 2009-126 is derived from PB from the same patient; 2007-043 is derived from BM and 2007-047 is derived from PB from the same patient

ChIP-seq data was then confirmed at the endogenous level in leukemic cell lines and primary patient samples, as well as in healthy human CD34^+^ stem/progenitor cells. As shown in Fig. 2a, both endogenous HIF1 and HIF2 binding to glycolysis-related genes was observed in K562 cells grown under hypoxia. Also, endogenous HIF binding to glycolysis-related genes could be induced when cells were stimulated with DMOG under normoxic conditions to stabilize HIF transcription factors, albeit to different levels as compared to cells that were grown under hypoxia. In healthy CB CD34^+^ cells, a strong HIF1 binding to glycolysis-related loci was also observed, where HIF2 binding was not detected, suggesting that in normal cells it is particularly HIF1 that exerts control over glycolysis genes (Fig. 2b).

In primary patient AML CD34^+^ cells, we observed that both HIF1 and HIF2 associated with glycolysis-related loci, although patient-specific differences were also noted, whereby HIF1 binding was more dominant than HIF2 in some cases (Fig. 2c, d). We compared HIF binding in primary AML CD34^+^ cells derived from the hypoxic bone marrow environment and compared that to more normoxic peripheral blood-derived AML CD34^+^ but in two tested cases comparable results were obtained (Fig. 2e).

We also wished to compare ChIP efficiencies using endogenous and tagged HIF approaches. We overexpressed HIF1 and HIF2 EGFP fusion proteins (in K562 cells) with an empty EGFP expressing vector as control. The cells were sorted for EGFP expression and incubated under normoxia or hypoxia (24 h) as indicated (Additional file 3: Figure S3a). ChIP-QPCR was performed using antibodies against EGFP (recognizing HIF:EGFP fusions), and HIF1 and HIF2 (recognizing HIF:EGFP fusions as well as endogenous HIF). As shown in Additional file 3: Figure S3a, the amount of HIF:EGFP fusions was approximately equal on a common HIF locus (ALDOA) when precipitated by αEGFP antibodies (green bars). Precipitation with antibodies against HIF1 and HIF2 resulted in comparable signals for the different antibodies (although with a slightly lower signal for HIF2), showing that the HIF antibodies have rather comparable affinities. However, the control group incubated under hypoxia (without HIF:EGFP over expression), shows an approximately tenfold higher HIF1 signal compared to HIF2, indicating that under these conditions the amount of chromatin-bound HIF1 is tenfold higher than the amount of HIF2. On the basis of these observations we conclude that our overexpression models result in HIF chromatin binding and transcriptional activities that are relatively comparable to the hypoxia-induced endogenous levels, whereby we also make note of the fact that our HIF2 models probably overestimate the true endogenous role under hypoxia in our cellular systems. No HIF was present on a non-binding locus (GATA5, Additional file 3: Figure S3b).

### Glycolysis but not TCA cycle or glutaminolysis-related genes are controlled by hypoxia and HIFs

**Fig. 3:**
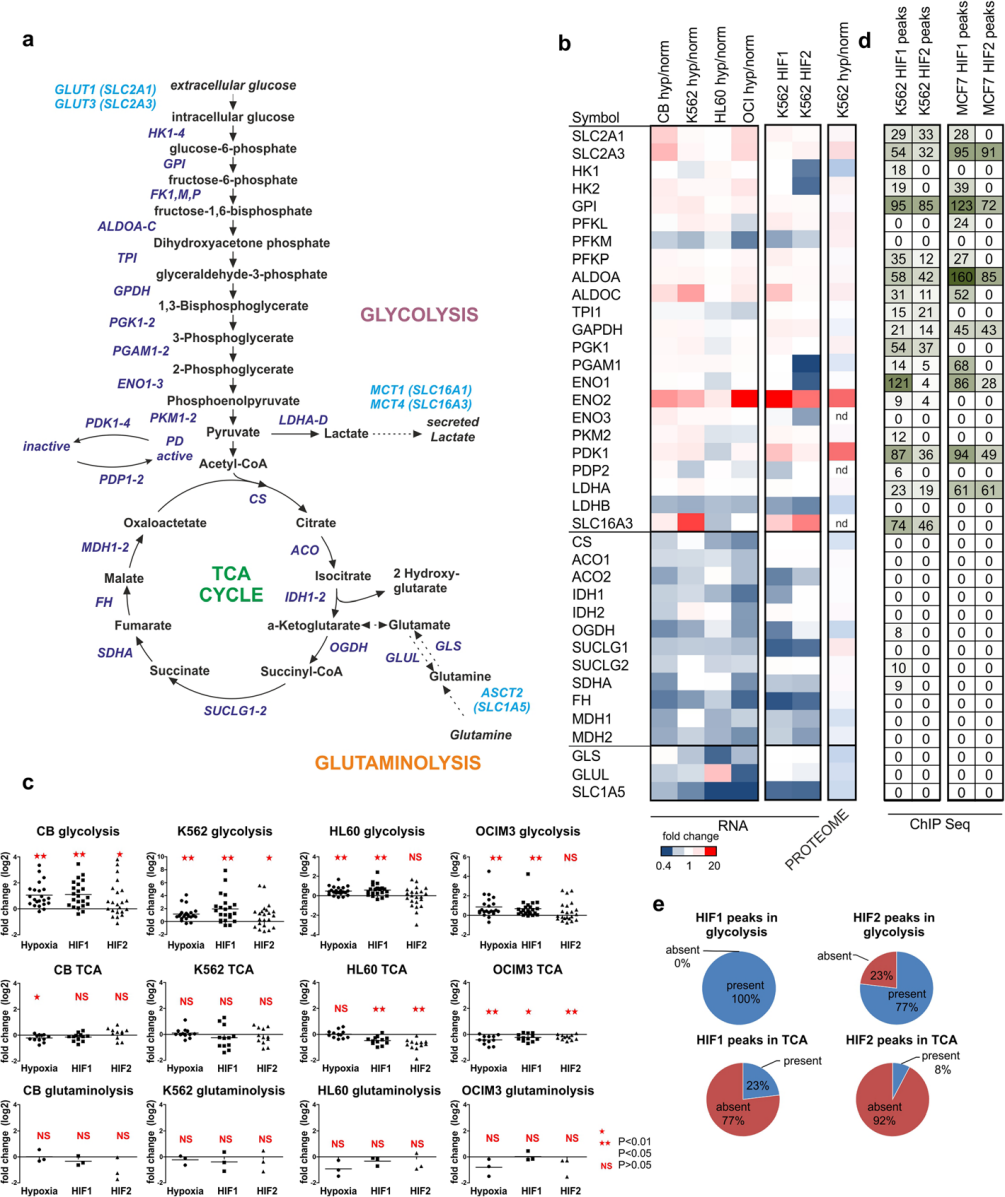
Glycolysis but not TCA activity is controlled by hypoxia and HIFs. **a** Schematic representation of glycolysis, TCA, and glutaminolysis pathways. **b** Hypoxia induces glycolysis but not TCA genes in normal CB CD34^+^ and leukemic K562, HL60, and OCI-AML3 cells. As comparison, transcriptome changes induced by overexpression of oxygen-insensitive HIF mutants in K562 cells grown under normoxia is shown in the last two columns. The last column shows the quantitative proteome data of K562 cells grown under hypoxia (24 h) or normoxia and the fold change in protein expression is shown. **c** Transcriptome changes in CB CD34^+^ cells and various leukemic cell lines upon overexpression of HIF1(P402A, P564A)-EGFP and HIF2(P405A, P531A). Transcriptome data is shown as fold change over controls. **d** ChIPseq data showing binding to glycolysis but not TCA loci. Peak heights are shown. For comparison, we also plotted peaks heights for HIF1 and HIF2 binding to glycolysis and TCA-related genes in MCF7 cells published by Schödel et al [47]. **e** Pie charts showing the relative binding of HIF1 and HIF2 to glycolysis-related loci and TCA-related loci. When at least one isoform of each enzyme was bound by HIFs at each consecutive step in these pathways then the total was 100%, as was seen for HIF1 bound to glycolysis-related loci

Since ChIP-seq and transcriptome studies indicated that HIFs in particular control glycolysis but not other metabolism-associated processes, we wished to extend our analysis to other cellular systems and generate a comprehensive detailed map of direct HIF binding and transcriptional control over all enzymes mediating glycolysis, TCA cycle and glutaminolysis (Fig. 3a). First, we performed genome-wide transcriptome studies across a panel of human leukemic cells lines as well as in normal CB CD34^+^ stem/progenitor cells upon culturing under hypoxia. Only glycolysis-related genes were upregulated under hypoxia, while for TCA cycle or glutaminolysis-related genes no difference or in some cell lines even a general decrease in expression was observed (Fig. 3b). Interestingly, cell type-specific differences were also noted in the hypoxia-induced changes in glycolysis-related genes, for instance in the case of glucose importers (SLC2A1, SLC2A3), PFKL and the lactate exporter SLC16A3, suggesting that depending on the genetic background cells respond differently. Similar to culturing under hypoxia, expression of oxygen-insensitive HIF1α(P402A,P564A)-EGFP or HIF2α(P405A,P531A)-EGFP consistently induced upregulation of glycolysis-related genes but not TCA cycle or glutaminolysis-related genes, both in normal CB CD34+ cells as well as across a panel of leukemic cell lines (Fig. 3b, 3c). We also performed quantitative proteome analyses in K562 cells grown under hypoxia for 24 h and these studies further confirmed upregulation of glycolysis-related genes at the protein level (Fig. 3b).

We then analyzed the level of direct HIF binding to glycolysis, TCA cycle, and glutaminolysis-related genes and observed that in fact almost all promoters of glycolysis-related genes were bound by HIF1, while for TCA cycle-related genes this was only 23% (Fig. 3d, e). Similarly, we observed that 77% of the promoters of glycolysis-related genes were bound by HIF2, while for TCA cycle-related genes this was only 8% (Fig. 3d, e). The strongest HIF1 binding was observed to ENO1, followed by GPI1, PDK1, SLC16A3, ALDOA, SLC2A3, PGK1 and PFKP, and for HIF2 similar binding profiles were seen except for ENO1 where binding was significantly weaker compared to HIF1 (Fig. 3d). For comparison, we also plotted peak heights for HIF1 and HIF2 binding to glycolysis and TCA related genes in MCF7 cells published by Schödel et al. [47].

**Fig. 4:**
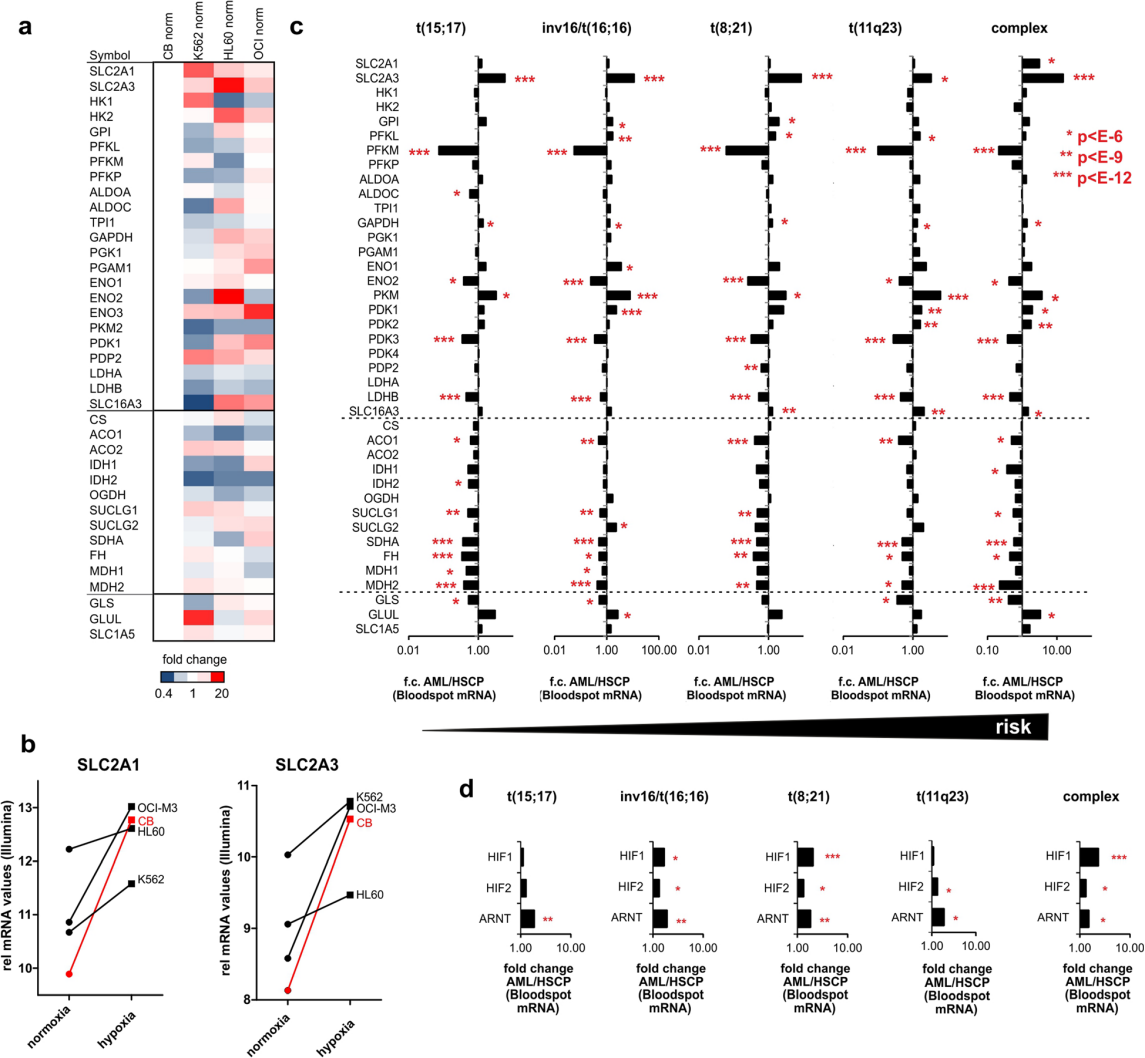
Leukemic cells adopt hypoxia like glycolytic signaling under normoxic conditions and various glycolytic genes are upregulated across multiple AML subtypes. **a** A number of glycolysis and TCA genes are upregulated in leukemia cell lines compared to normal CB CD34^+^ cells under normoxic conditions. A heatmap of gene array data is shown whereby expression levels in CB CD34^+^ cells were set to 1. **b** Expression of SLC2A1 and SLC2A3 under normoxic and hypoxic conditions in normal CB CD34^+^ cells and leukemia lines. **c** Expression of glycolysis, TCA, and glutaminolysis genes in primary AML patient samples. Data were taken from the bloodspot database. **d** Expression of HIF1, HIF2, and ARNT in primary AML patient samples

Since cancer cells have been suggested to be intrinsically glycolytic, and also since we previously noted that human CD34^+^ cells expressing various oncogenes have been described to express hypoxic gene signatures even when cultured under normoxic conditions [41], we questioned whether at baseline under normoxic conditions the expression of glycolysis, TCA cycle or glutaminolysis-related genes would be different between normal CD34^+^ stem/progenitor cells and leukemic cells. We noted a consistent upregulation of glucose importers SLC2A1 and SLC2A3 in leukemic cells, but also various other glycolysis-related genes were upregulated in leukemia although variation between different cell lines was noted as well (Fig. 4a, b). In fact, also various TCA cycle-related genes were higher expressed in leukemias compared to normal CD34^+^ cells, and the same was seen for some glutaminolysis-related genes. In particular, K562 cells showed an upregulation of the glutamine importer SLC1A5 and the glutamine-to-glutamate converting enzyme GLUL (Fig. 4a), in line with previous data showing that overexpression of BCR-ABL in human CD34^+^ cells as well as primary CML patient samples display enhanced glutaminolysis [41].

We further investigated expression of glycolysis-related genes in primary AML patient samples (taken from the Bloodspot database [49]) compared to normal stem/progenitor cells. As shown in Fig. 4c, a number of glycolysis genes were consistently upregulated in primary AML cells representing different risk categories, and in particular an upregulation of SLC2A3, PKM, PDK1, PDK2 and SLC16A3 was noted. TCA-related genes were typically downregulated in AML compared to normal stem/progenitors, while GLS was downregulated and GLUL upregulated (Fig. 4c). A modest but significant upregulation of HIF1, HIF2, and ARNT was observed as well in AML (Fig. 4d).

### Generation of CRISPR/Cas9-mediated specific HIF1, HIF2, and ARNT knockout lines

**Fig. 5:**
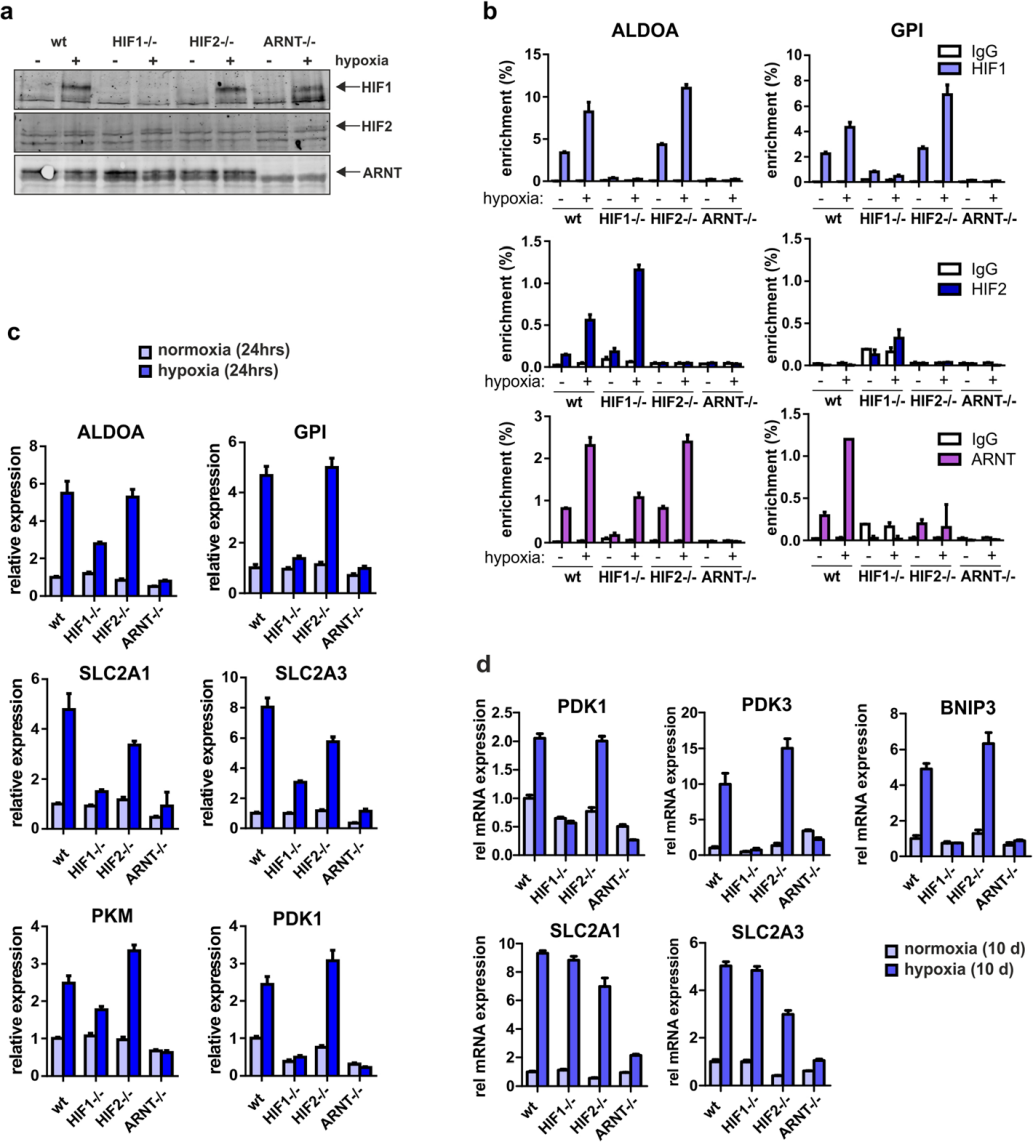
Generation of CRISPR/Cas9-mediated knockout K562 lines for HIF1, HIF2, and ARNT. **a** Single-cell-derived knockout lines were generated and validated by sequencing after which 4 single cell-derived lines were pooled for further analyses. Cells were grown for 24 h under hypoxia and extracts were western blotted for the presence of HIF1, HIF2, and ARNT. **b** K562 cells were treated as in **a**, and ChIP Q-PCR experiments were performed. The knockout is shown below the *x*-axis, the antibodies used for ChIP-PCRs are shown in the colored boxes (HIF1 in top panels, HIF2 in middle panels, ARNT in lower panels), and the loci to which binding is investigated is shown at the top (ALDOA for left panels and GPI for right panels). **c** Q-PCR was performed on knockout lines. Cells were grown under hypoxia for 24 h. **d** Experiment as in **c** but now cells were grown under hypoxia for 10 days to evaluate gene expression changes under chronic hypoxia conditions

In order to functionally study the role of HIF transcription factors in controlling glycolysis under hypoxia, we generated specific HIF1, HIF2 and ARNT knockout K562 lines using a CRISPR/Cas9 approach. Several single cell-derived knockout lines were generated and the introduction of loss-of-function mutations was validated by Sanger sequencing (Supplementary Materials and Methods). Multiple validated single cell-derived clones (typically 4) were then again combined in order to rule out individual clone-specific phenotypes, and all data was generated using these pooled lines. Western blot using antibodies against HIF1α, HIF2α and ARNT was performed to confirm knockout of the respective genes (Fig. 5a). In order to functionally validate our CRIPR/Cas9 lines, we performed ChIP-PCRs using antibodies against endogenous HIF1, HIF2, or ARNT both under normoxia as well as under hypoxia. Several loci were investigated and representative data for ALDOA and GPI are shown in Fig. 5b. A clear induction of HIF1, HIF2, and ARNT binding was observed under hypoxia in wild type (wt) K562 cells. Specific loss of HIF1 binding was observed in HIF1^−/−^ lines, specific loss of HIF2 binding was observed in HIF2^−/−^ lines, and no HIF1 or HIF2 binding was observed in ARNT^-/-^ lines, as expected (Fig. 5b). In the absence of either HIF1 or HIF2, some ARNT binding was still observed on the ALDOA locus, indicating that expression of either one of these HIF factors is sufficient to recruit ARNT to the chromatin.

Next, we questioned whether the expression of glycolysis genes would be affected upon loss of HIF signaling. In initial experiments, cells were grown under hypoxia for 24 h after which RNA was isolated for analyses. As shown in Fig. 5c for 6 examples, knockout of ARNT strongly impaired the hypoxia-induced upregulation of these genes. We also performed a quantitative proteome analyses on either wt cells or ARNT^−/−^ K562 cells grown under normoxia or under hypoxia for 24 h. As depicted in Additional file 4: Figure S4a, the hypoxia induced upregulation of glycolysis-related proteins was largely HIF-dependent. In order to determine whether glycolysis-related genes could be re-expressed in our CRISPR knockout lines, we re-introduced the oxygen-insensitive HIF1 and HIF2 mutants in the HIF1^−/−^ or HIF2^−/−^ K562 cells, respectively. Next, we isolated mRNA from cells grown under hypoxia or normoxia for 24 h, performed Q-RT-PCRs, and show that re-expression of HIFs results in elevated expression of glycolysis-related genes (Additional file 4: Figure S4b).

Remarkably, loss of HIF1 had a much stronger impact on the hypoxia-induced upregulation of glycolysis genes as compared to HIF2 loss, suggesting that under these early acute hypoxic stress conditions, HIF1 is more important to control expression of these genes. To investigate this further, we also analyzed mRNA from cells that had been grown under chronic hypoxia conditions for a period of 10 days. Loss of ARNT still impaired the hypoxia-induced upregulation of several glycolysis genes as well as the HIF target BNIP3. For the glucose transporters SLC2A1 and SLC2A3, a clear compensatory effect was noted whereby both HIF1 or HIF2 could drive expression of these genes together with ARNT under hypoxia (Fig. 5c), although other HIF-independent compensatory mechanisms might play a role as well. In contrast, hypoxia-induced expression of PDK1 and PDK3 and also BNIP3, remained rather dependent of HIF1 specifically (Fig. 5d).

### Loss of HIF signaling does not impact on proliferation or the metabolic state of cells under hypoxia

**Fig. 6:**
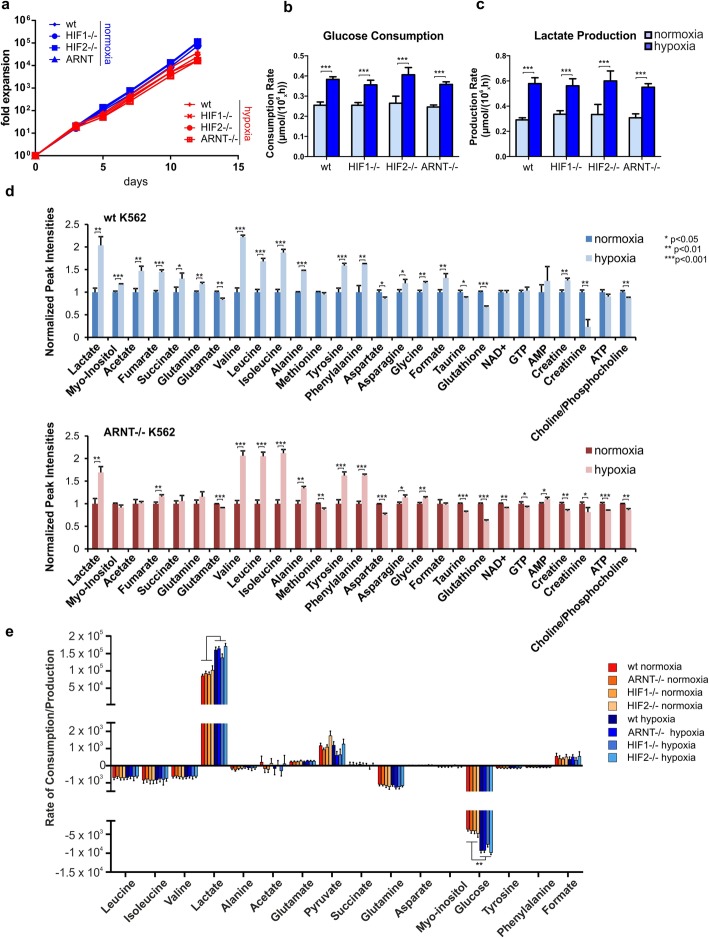
Loss of HIF signaling does not impair hypoxia-induced glycolysis. **a** Growth curves of K562 HIF1, HIF2, and ARNT knockout cells under hypoxia and normoxia. **b**–**c** Glucose consumption (**b**) and lactate production (**c**) of K562 HIF1, HIF2, and ARNT knockout cells grown under normoxic and hypoxic growth conditions for 24 h. *p < 0.05. **d** 1D 1H-NMR extract metabolite intensities from K562 and ARNT knockout cells grown under hypoxia or normoxia for 24 h. **e** 1D 1H-NMR medium metabolite intensities from K562 wildtype (wt) and ARNT knockout cells grown under hypoxia and normoxia with medium collection at 18, 21, and 24 h for analysis to calculate the rate of production/consumption of indicated metabolites

In order to functionally study the cell biological consequences for loss of HIF signaling under hypoxia, a number of studies were undertaken. First, cell proliferation was investigated and a slight reduction in proliferation rate was observed when cells were grown under chronic hypoxia, but surprisingly the absence of HIF signaling did not impact on the proliferation rate (Fig. 6a). When cells were plated and grown under higher cell densities (starting at 0.1 × 10^6^ cells per ml), proliferation was more strongly reduced under hypoxia conditions but also under those conditions loss of HIF signaling did not impact at all on the proliferation rate (data not shown). In order to validate whether our cells adopted a glycolytic metabolic state under hypoxia we determined glucose consumption and lactate production levels by spectrophotometric enzyme assays. As shown in Fig. 6b,c, both glucose consumption and lactate production were increased upon culturing under hypoxia, as expected, but surprisingly knockout of HIF1, HIF2 or ARNT did not impact on the glycolytic state of cells at all. This occurred independent of whether cells were analyzed under acute hypoxic stress conditions (24 h, Fig. 6b,c) or under chronic hypoxia conditions (day 10, Additional file 5: Figure S5a). In order to determine whether these observations would be specific for leukemic cells, healthy CB-derived CD34^+^ cells were transduced with shRNA lentivectors to downregulate ARNT (Additional file 5: Figure S5b), after which cells were plated under normoxia or hypoxia. Again, no impact on cell proliferation was noted (data not shown). And while hypoxia nicely induced a shift towards a more glycolytic metabolic state, the loss of HIF signaling did not impact on the level of glucose consumption or lactate production under acute or chronic hypoxic conditions (Additional file 5: Figure S5b). This was despite an efficient knockdown of ARNT and consequently a loss of HIF-mediated upregulation of glycolytic genes like ALDOC and PDK1 upon hypoxic growth (Additional file 5: Figure S5c).

Since these findings were unexpected, we referred to alternative methods to study the metabolic state of cells and performed 1D-NMR studies in order to quantify several intracellular and extracellular metabolites in K562 HIF1, HIF2, and ARNT knockout cells grown under hypoxia or normoxia. While several intracellular metabolites changed upon culturing under hypoxia, including an increase in lactate production, the loss of HIF1, HIF2, or ARNT did not impact on most of these hypoxia-induced changes (Fig. 6d, Additional file 6: Figure S6). Besides changes in lactate production, several other intracellular metabolites were affected by hypoxia, most notably significant reductions in the antioxidant glutathione, which is required to detoxify reactive oxygen species. Also significant reductions in phosphocholine/choline were noted, suggesting that phosphatidylcholine synthesis via the Kennedy pathway is affected [50]. Various changes in intracellular amino acids were noted as well, including increases in the essential branched-chain amino acids leucine, isoleucine and valine under hypoxia. However, none of these levels were affected by loss of HIF1, HIF2, or ARNT. The only consistent changes we noted were that the hypoxia-induced increases in intracellular myo-inositol, formate, and acetate were lost upon knockout of HIF signaling, while the hypoxia-induced reduction in intracellular creatinine was not as pronounced in the absence of HIFs (Fig. 6d, Additional file 6: Figure S6).

We also performed time course 1D-NMR on the medium in which cells were grown at several time points, either under normoxia or hypoxia. Over the time course of the experiments, cells consumed significant amounts of glutamine, the branched amino acids leucine, isoleucine, and valine, but only the consumption of glucose was significantly enhanced by hypoxia (Fig. 6e). Reversely, a significant increase in lactate production was observed under hypoxia, but in line with our previous data, the absence of HIFs did not impact on the hypoxia-induced glycolytic state (Fig. 6e).

**Fig. 7:**
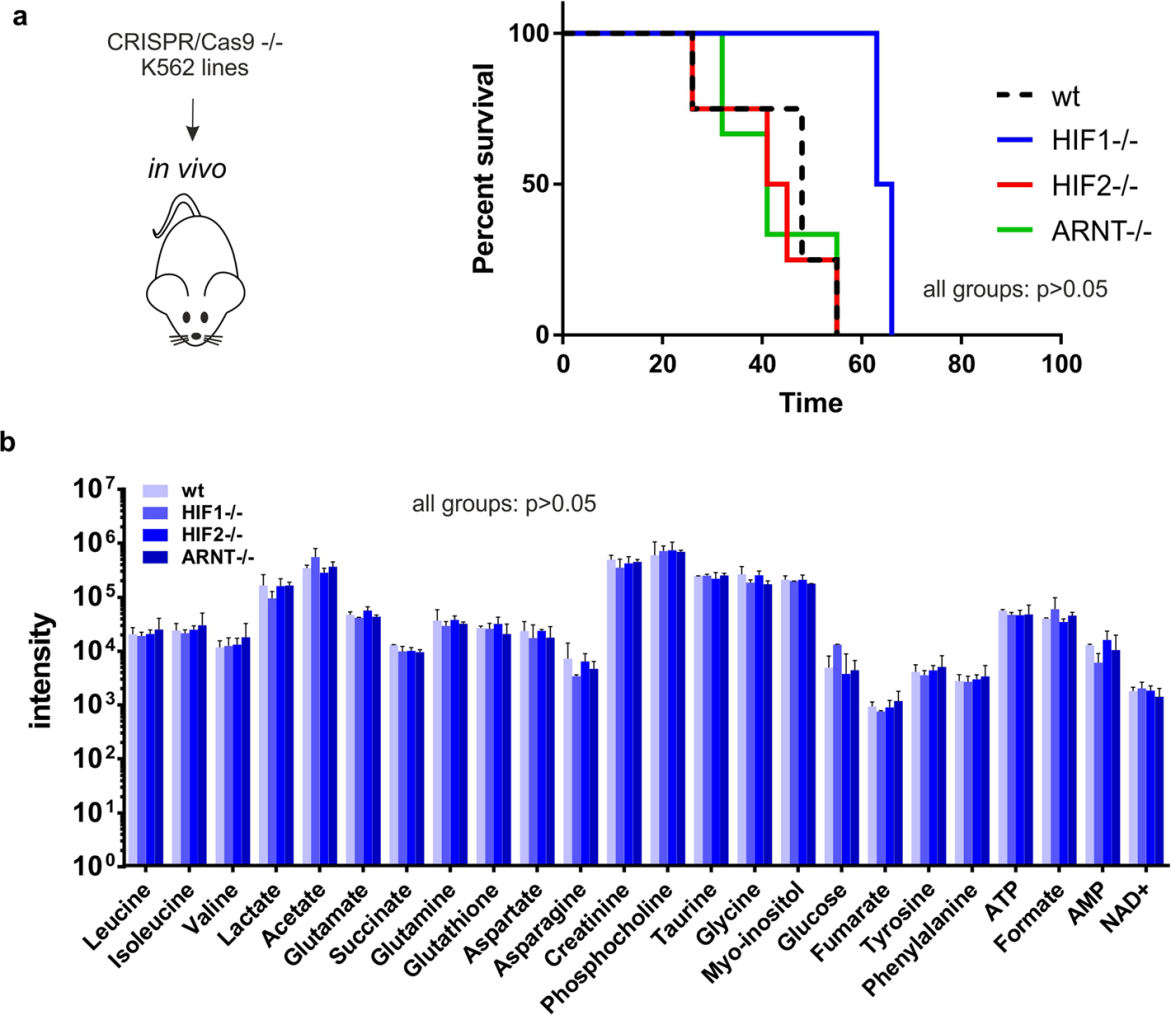
Loss of HIF signaling does not impair leukemia development in vivo. **a** K562 wt, HIF1, HIF2, and ARNT (*n* = 5) knockout cells were injected into sublethally irradiated NSG mice (*n* = 5 per group) and leukemia development was monitored. No significant differences in overall survival were observed. **b** Tumors (wt *n* = 2, HIF1 *n* = 2, HIF2 *n* = 4, gARNT *n* = 2) were harvested from leukemic mice and were subjected to 1D 1H-NMR analyses in order to quantify intracellular metabolite levels

In order to evaluate whether loss of HIF signaling would impact on tumor development in vivo, we injected our CRISPR-Cas9 knockout cells into immunodeficient NSG mice (*n* = 5 per group). As shown in Fig. 7a, no effects were observed on the latency of tumor development upon loss of either HIF1, HIF2, or ARNT. Some mice did not develop tumors at all, in line with the notion that K562 cells do not engraft well in some transplanted animals. We did note a slight trend towards a latency of leukemia onset in HIF1^−/−^ animals but this did not reach significance and was also not noted in mice transplanted with ARNT^−/−^ cells, in which HIF signaling is completely absent. Furthermore, we quantified several intracellular metabolites from extracted tumor cells by 1D-NMR studies and these results also did not reveal differences in glycolysis upon loss of HIF signaling in vivo (Fig. 7b).

### Inhibition of BCR-ABL tyrosine kinase activity does impair glycolysis independent of hypoxia and HIF signaling

**Fig. 8:**
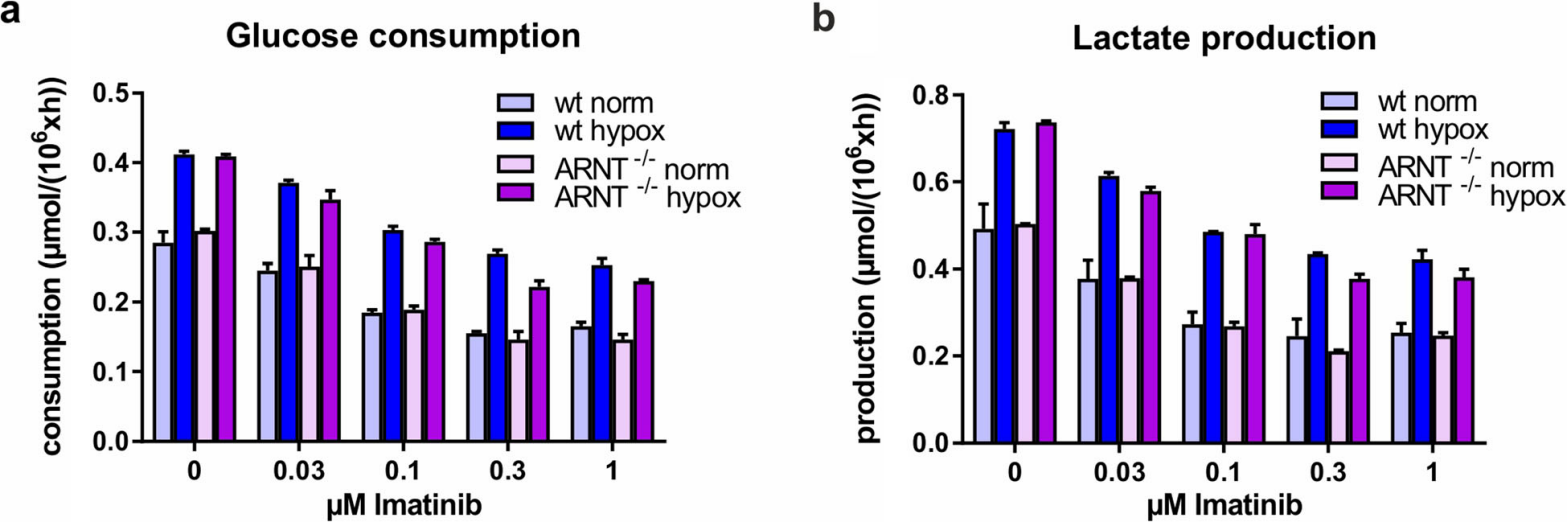
Inhibition of BCR-ABL does impact on the glycolytic state of K562 cells, independent of HIF signaling and hypoxia. Glucose consumption (**a**) and lactate production (**b**) of K562 wt and ARNT knockout cells grown under normoxic and hypoxic growth conditions for 24 h cultured in the presence of increasing doses of imatinib

Next, we questioned whether inhibition of signaling networks downstream of the oncogene BCR-ABL would impact on glycolysis. We performed glucose consumption and lactate production assays in K562 cells treated with increasing doses of imatinib. In contrast to loss of HIF signaling, inhibition of BCR-ABL kinase activity did result in reduced glycolysis in a dose-dependent manner (Fig. 8a and b). While culturing under hypoxia resulted in enhanced glycolysis as expected, a comparable reduction in glucose consumption and lactate production upon Imatinib treatment was observed under normoxic and hypoxic conditions, and also loss of HIF signaling as a consequence of ARNT knockout did not impact on the glycolytic state (Fig. 8a, b). These data clearly indicate that BCR-ABL mediated control over glycolysis occurs independently of hypoxic signaling modules.

## Discussion

In the current study, we aimed to obtain a deeper insight into the roles of HIF transcription factors in human hematopoietic stem and progenitor cells and their leukemic counterparts. While we initially hypothesized that various metabolic features would particularly be under the control of HIFs, we conclude that even though the vast majority of the glycolysis-related loci are directly regulated by HIFs under hypoxia, this control is not strictly required to adopt a glycolytic metabolic state under acute or chronic hypoxic conditions.

We carefully mapped all genomic loci that are directly bound by either HIF1 or HIF2 in human hematopoietic leukemic cells and linked this to transcriptional changes induced under hypoxia and this provided a comprehensive view on their potential cell biological roles. As expected, we identified known targets like VEGFA and VEGFB as loci that were directly bound and strongly upregulated by both HIF1 and HIF2 (Additional files 8 and 9: Tables S1 and S2) [9, 28, 29]. Less expected was the observation that HIFs can also directly control various chromatin modifying enzymes. This also included a number of histone methyltransferases, deacetylases and demethylases, in line with data published previously in liver cells [51], leaving open the possibility that the epigenome might change under hypoxic conditions, in part via HIF signaling, a notion that will be further investigated. But among the most strongly bound and upregulated HIF loci, we identified genes that were predominantly associated with the glycolytic metabolic program. This included for instance PFKFB4, which is a bifunctional kinase/phosphatase that regulates the concentration of the glycolytic by-product fructose-2,6-bisphosphate (F2,6BP) [52]. We did not find evidence for HIF-exerted transcriptional regulation control over the Krebs cycle enzymes, the pentose phosphate pathway, gluconeogenesis, or the glutaminolysis pathway, and in fact many of these genes were down regulated under hypoxia or upon overexpression of HIF mutants, both in our model systems as well as in primary AML patient samples. In contrast, we did observe a direct control of HIFs over virtually all metabolic steps of the glycolytic pathway, in line with previously published data in breast and liver cancer cell lines [47, 48, 51]. Multiple homologues of enzymes exist at various levels of this pathway that are considered to be able to mediate the glycolytic flux, as is for instance the case for the glucose transporters SLC2A1 and SLC2A3, and the enolases ENO1 and ENO2 and all of these were strongly bound and upregulated by HIFs. At the level of phosphofructokinase, it was particularly PFKL and PFKP but not PFKM that was under HIF control, in line with what we observed in primary AML patient samples. Some specificity in HIF signaling was also observed, and for instance at the level of the hexokinases, we observed that HK1 and HK2 were exclusively controlled by HIF1 and not HIF2.

These data suggested that HIFs would not merely induce a glycolytic cellular state by controlling very specific rate-limiting steps of glycolysis but rather that it is ensured that all components of the complete pathway remain expressed at sufficient levels under hypoxic conditions. We then further challenged this notion by functional studies in which we knocked out HIF signaling using a CRIPSR-Cas9 approach. In the complete absence of HIF signaling, by knocking out HIF1, HIF2 or the essential co-factor ARNT, we observed that the hypoxia-induced upregulation of glycolysis-associated genes was almost completely abrogated. Surprisingly, however, no effects were seen on the hypoxia-induced increase in glucose consumption or lactate production as determined by various assays at both the intracellular and extracellular level, under acute and more chronic hypoxia conditions, both in normal CD34^+^ cells as well as in leukemic cells. These data clearly contradict earlier suggestions that HIFs would be critical mediators of the glycolytic state. However, while Simsek et al. showed that LT-HSCs utilize glycolysis instead of mitochondrial oxidative phosphorylation, coinciding with an enhanced expression of HIF1, they did not show that HIF1 would be strictly required to maintain the glycolytic state [8]. Takubo et al. utilized Mx1-CRE-inducible knockout mouse models and showed that LT-HSC quiescence depended on HIF1 expression, coupled to the capacity for serial transplantation, and while an increase in mitochondrial activity was noted, the effects on glycolysis were not investigated [12]. In another study, making use of the same inducible mouse model, they did study glucose uptake in various stem and progenitor cell compartments. However, they did not see significant changes in glucose uptake in any of these compartments in the absence of HIF signaling, although LDH activity and lactate production under hypoxia was slightly decreased [31]. Miharada et al. showed that HIF1 controls the number of GPR78^+^ HSCs, and that CRIPTO-GPR78 signaling is required for HSC maintenance in the endosteal niche and HSC quiescence, but again no direct link between HIF signaling and glycolysis was shown [13]. Studies in Hif1^−/−^ murine embryonic fibroblasts (MEFs) have indeed shown that cells die due to an excess of ROS production, presumably due to a failure of making the switch from oxidative phosphorylation to glycolysis [53]. But glycolytic activity was not investigated in that study, and moreover hematopoietic cells do not die under hypoxia conditions suggesting that cell-specific roles must exist. Taken together, while various papers indeed indicate that HIFs can somehow control the expression of glycolytic genes in various cell types (as also nicely reviewed in [15]), most of these do not provide functional data in the hematopoietic stem/progenitor cell compartment.

Possibly, the HIF-mediated control over glycolysis genes under hypoxia acts as a safeguarding mechanism, but is not strictly required to maintain a glycolytic state. Obviously, many other signaling networks and transcription factors can act on the promoters and enhancers that drive the expression of glycolysis genes, including MYC, STAT3/5, and the PI3K pathway, and even in the absence of HIF signaling, such pathways are apparently sufficient to maintain glycolytic activity at high enough levels under hypoxia. We initially hypothesized that particularly in cancer cells, which display a hyperactivation of various pathways such as those described above would be less dependent on HIFs to maintain a glycolytic state. However, also in normal CB CD34^+^ cells we observed that knockdown of HIF signaling did not affect the hypoxia-induced glucose uptake and lactate production, both under acute and chronic hypoxia conditions, although it must be noted that these culture conditions include relatively high cytokine concentrations that would also induce strong activation of MYC, STAT3/5 and PI3K pathways. Indeed, when we analyzed K562 Encode ChIP-seq datasets we observed strong binding of STAT5 and MYC to the promoters of glycolysis genes like SLC2A1, SLC2A3, HK1, PKM (data not shown), indicating that loci are under control of various pathways. Upon treatment of K562 cells with imatinib, we observed clear reductions in glucose consumption and lactate production both under normoxic and hypoxic conditions, indicating that BCR-ABL can impact on the glycolytic state of cells independently of hypoxia-induced HIF signaling. Further studies will be needed to determine whether real differences exist in the HIF dependency of normal versus leukemic cells, but our data clearly challenge the view that HIFs would act as oncogenes merely by imposing a glycolytic state on cancer cells. It is quite likely that the conditions under which cells are studied will have a main impact on the exact role of HIFs, and might dictate whether they act as tumor suppressors or oncogenes, as recently extensively discussed in various papers, but what is clear now is that they do not simply act as gatekeepers of glycolysis.

Recently, it was shown that HIF2 is critically important for the maintenance of both normal as well as leukemic human hematopoietic stem/progenitor cells, whereby loss of HIF2 triggered an apoptotic response via activation of the unfolded-protein response pathway [20], indicating that glycolysis-independent mechanisms might be important downstream of HIFs that control hematopoietic stem cell fate. We find that EGR1, which acts as a hematopoietic stem cell self-renewal factor is also directly controlled by hypoxia, although this occurred in a completely HIF-independent manner. Loss of EGR1 results in loss of HSC quiescence and spontaneous mobilization [54]. EGR1 has been shown to promote hypoxia-induced autophagy [55], and we and others recently showed that autophagy is important to maintain HSC [56–58]. Furthermore, we observed that specifically HIF1 but not HIF2, was able to bind and drive BNIP3 expression, in line with data published by Sowter et al [59]. BNIP3 interacts with processed LC3 at phagophore membranes to promote sequestration of mitochondria within the autophagosome for degradation [59, 60]. Another HIF1-specific set of target genes was associated with splicing, possibly in line with published data indicating that alternative splicing may occur under hypoxia, generally promoting exon inclusion for hypoxia-induced genes, which included PDK1 [61]. Together, these data highlight the multitude of mechanisms via which HIFs might contribute to stem cell fate in normal and cancer cells, whereby their role in controlling glycolysis appears to be less pronounced.

## Conclusions

We have characterized the chromatin-binding profiles of HIF1 and HIF2 in human leukemic cells in detail and correlated that with transcriptional changes and conclude that these transcription factors can transactivate all enzymes that participate in the glycolytic pathway while OxPHOS- or glutaminolysis-related enzymes are not controlled by HIFs. Surprisingly, however, complete loss of HIF signaling via CRISPR/Cas9-mediated knockout of HIF1, HIF2, or ARNT did not at all impact on glucose consumption or lactate production in leukemic cells, neither in vitro nor in vivo after transplantation of knockout cells to immune deficient xenograft mice. Also, the hypoxia-induced glycolytic state of healthy CD34^+^ cells was not affected upon knockdown of HIF1 or HIF2. In contrast, inhibition of BCR-ABL did impact on glucose consumption and lactate production regardless of the presence of HIFs. These data indicate that oncogene-mediated control over glycolysis can occur independently of hypoxic signaling modules.

## Supplementary information

**Additional file 1:** **Figure S1.** Identification of HIF1 and HIF2 chromatin binding sites in human leukemic cells. a. About 2/3 of HIF1-bound or HIF2-bound loci localize close to TSSs and 1/3 do not. b. Some HIF1/2 bound loci distal form TSSs potentially regulate lncRNAs. An example screenshot of a lncRNA potentially controlled by HIFs is shown on the right. c. HIF binding to Chromatin modifying enzymes defined by GO RP. d. Overlap in ChIPseq targets in K562 versus MCF7 cells. e. GO reactome pathways in common HIF targets across different cell types.

**Additional file 2:** **Figure S2.** Identification of co-occuring transcription factor motifs at HIF1 and HIF2-bound loci. A transcription factor (TF) motif screen was performed to identify co-occurring TF motifs at HIF1 (a) and HIF2 (b) loci. The overlap is shown in c. Some representative screens shots are shown in d. NRF1, ELK1, H3K4me3, H3K27ac and H3K27me3 tracks from K562 cells were retrieved from ENCODE.

**Additional file 3:** **Figure S3.** We overexpressed HIF1 and HIF2 EGFP fusion proteins (in K562 cells) with an empty EGFP expressing vector as control. The cells were sorted for EGFP expression and incubated under normoxia or hypoxia (24 hrs) as indicated. ChIP-QPCR was performed using antibodies against EGFP (recognizing HIF:EGFP fusions), and HIF1 and HIF2 (recognizing HIF:EGFP fusions as well as endogenous HIF). b. No HIF was present on the GATA5 locus to which HIFs do not bind.

**Additional file 4:** **Figure S4.** a. Quantitative proteome data of wt or ARNT^-/-^ K562 cells grown under hypoxia (24 hrs) or normoxia and protein expression levels are shown for glycolysis, TCA and glutaminolysis-related proteins. The K562 wt normoxia/hypoxia data are identical to those used for Figure 3b (where fold changes are shown rather than relative protein expression levels), but are again shown here to compare with ARNT^-/-^ cells. b, Rescue experiment by re-introducing the oxygen-insensitive HIF1 and HIF2 mutants in the HIF1^-/-^ or HIF2^-/-^ K562 cells, respectively. Q-RT-PCRs were performed indicating that glycolysis-related genes can be re-expressed upon overexpression of HIF mutants.

**Additional file 5:** **Figure S5.** Glucose consumption and lactate production in CB CD34^+^ and K562 cells. a. Glucose consumption (left panel) and lactate production (right panel) of K562 cells, grown for 10 days under hypoxia (1% O2). *: P<0.05, n.s.: Non-significant. b. Glucose consumption (left panel) and lactate production (right panel) of cordblood. CD34^+^ cells after 24 hour hypoxia (1% O2), with knockdown of ARNT. c. Knockdown efficiency of ARNT (left panel) and expression of target genes (middle and right panel) in cordblood CD34^+^ cells. *: P<0.05, n.s.: Non-significant.

**Additional file 6:** **Figure S6.** 1D-NMR extract metabolite intensities from K562 HIF1 and HIF2 knockout cells grown under hypoxia or normoxia for 24 hr. The K562 wt data is identical to that depicted in Fig. 6b but was added here again for reference.

**Additional file 7.** Supplemental Methods.

**Additional file 8:** **Supplemental Table 1.** ChIPseq data.

**Additional file 9:** **Supplemental Table 2.** transcriptome data.

## Data Availability

All ChIP-seq data is deposited at GEO under GSE123461.

## Abbreviations

AML: Acute myeloid leukemia
CB: Cord blood
ChIP: Chromatin immunoprecipitation
GFP: Green fluorescent protein
GPCR: G-protein coupled receptor
GSEA: Gene set enrichment analyses
HIF: Hypoxia induced factor
HSC: Hematopoietic stem cell
LSC: Leukemic stem cell
MEFs: Murine embryonic fibroblasts.
OXPHOS: Oxidative phosphorylation
ROS: Reactive oxygen species
TCA: Tricarboxylic acid cycle
TF: Transcription factor

## References

[1] Calvi Laura M., Link Daniel C. (2015). The hematopoietic stem cell niche in homeostasis and disease. Blood.

[2] Hoggatt Jonathan, Kfoury Youmna, Scadden David T. (2016). Hematopoietic Stem Cell Niche in Health and Disease. Annual Review of Pathology: Mechanisms of Disease.

[3] Parmar K., Mauch P., Vergilio J.-A., Sackstein R., Down J. D. (2007). Distribution of hematopoietic stem cells in the bone marrow according to regional hypoxia. Proceedings of the National Academy of Sciences.

[4] Mohyeldin A, Garzon-Muvdi T, Quinones-Hinojosa A (2010). Oxygen in stem cell biology: a critical component of the stem cell niche. Cell Stem Cell..

[5] Spencer Joel A., Ferraro Francesca, Roussakis Emmanuel, Klein Alyssa, Wu Juwell, Runnels Judith M., Zaher Walid, Mortensen Luke J., Alt Clemens, Turcotte Raphaël, Yusuf Rushdia, Côté Daniel, Vinogradov Sergei A., Scadden David T., Lin Charles P. (2014). Direct measurement of local oxygen concentration in the bone marrow of live animals. Nature.

[6] Mantel CR, O'Leary HA, Chitteti BR, Huang X, Cooper S, Hangoc G (2015). Enhancing Hematopoietic Stem Cell Transplantation Efficacy by Mitigating Oxygen Shock. Cell..

[7] Suda T, Takubo K, Semenza GL (2011). Metabolic regulation of hematopoietic stem cells in the hypoxic niche. Cell Stem Cell..

[8] Simsek T, Kocabas F, Zheng J, Deberardinis RJ, Mahmoud AI, Olson EN (2010). The distinct metabolic profile of hematopoietic stem cells reflects their location in a hypoxic niche. Cell Stem Cell..

[9] Keith B, Simon MC (2007). Hypoxia-inducible factors, stem cells, and cancer. Cell..

[10] Semenza Gregg L. (2012). Hypoxia-Inducible Factors in Physiology and Medicine. Cell.

[11] Schito L, Rey S, Konopleva M (2017). Integration of hypoxic HIF-alpha signaling in blood cancers. Oncogene..

[12] Takubo K, Goda N, Yamada W, Iriuchishima H, Ikeda E, Kubota Y (2010). Regulation of the HIF-1alpha level is essential for hematopoietic stem cells. Cell Stem Cell..

[13] Miharada K, Karlsson G, Rehn M, Rorby E, Siva K, Cammenga J (2011). Cripto regulates hematopoietic stem cells as a hypoxic-niche-related factor through cell surface receptor GRP78. Cell Stem Cell..

[14] Scortegagna M, Morris MA, Oktay Y, Bennett M, Garcia JA (2003). The HIF family member EPAS1/HIF-2alpha is required for normal hematopoiesis in mice. Blood..

[15] Semenza Gregg L. (2013). HIF-1 mediates metabolic responses to intratumoral hypoxia and oncogenic mutations. Journal of Clinical Investigation.

[16] Lee Kyoung Eun, Simon M Celeste (2012). From stem cells to cancer stem cells: HIF takes the stage. Current Opinion in Cell Biology.

[17] Rouault-Pierre K, Hamilton A, Bonnet D (2016). Effect of hypoxia-inducible factors in normal and leukemic stem cell regulation and their potential therapeutic impact. Expert Opin Biol Ther.

[18] Gao X N, Yan F, Lin J, Gao L, Lu X L, Wei S C, Shen N, Pang J X, Ning Q Y, Komeno Y, Deng A L, Xu Y H, Shi J L, Li Y H, Zhang D E, Nervi C, Liu S J, Yu L (2015). AML1/ETO cooperates with HIF1α to promote leukemogenesis through DNMT3a transactivation. Leukemia.

[19] Forristal C E, Brown A L, Helwani F M, Winkler I G, Nowlan B, Barbier V, Powell R J, Engler G A, Diakiw S M, Zannettino A C W, Martin S, Pattabiraman D, D'Andrea R J, Lewis I D, Levesque J P (2015). Hypoxia inducible factor (HIF)-2α accelerates disease progression in mouse models of leukemia and lymphoma but is not a poor prognosis factor in human AML. Leukemia.

[20] Rouault-Pierre Kevin, Lopez-Onieva Lourdes, Foster Katie, Anjos-Afonso Fernando, Lamrissi-Garcia Isabelle, Serrano-Sanchez Martin, Mitter Richard, Ivanovic Zoran, de Verneuil Hubert, Gribben John, Taussig David, Rezvani Hamid Reza, Mazurier Frédéric, Bonnet Dominique (2013). HIF-2α Protects Human Hematopoietic Stem/Progenitors and Acute Myeloid Leukemic Cells from Apoptosis Induced by Endoplasmic Reticulum Stress. Cell Stem Cell.

[21] Wang Yin, Liu Yan, Malek Sami N., Zheng Pan, Liu Yang (2011). Targeting HIF1α Eliminates Cancer Stem Cells in Hematological Malignancies. Cell Stem Cell.

[22] Koczula KM, Ludwig C, Hayden R, Cronin L, Pratt G, Parry H (2016). Metabolic plasticity in CLL: adaptation to the hypoxic niche. Leukemia..

[23] Vukovic Milica, Sepulveda Catarina, Subramani Chithra, Guitart Amélie V., Mohr Jasmine, Allen Lewis, Panagopoulou Theano I., Paris Jasmin, Lawson Hannah, Villacreces Arnaud, Armesilla-Diaz Alejandro, Gezer Deniz, Holyoake Tessa L., Ratcliffe Peter J., Kranc Kamil R. (2016). Adult hematopoietic stem cells lacking Hif-1α self-renew normally. Blood.

[24] Guitart Amelie V., Subramani Chithra, Armesilla-Diaz Alejandro, Smith Gillian, Sepulveda Catarina, Gezer Deniz, Vukovic Milica, Dunn Karen, Pollard Patrick, Holyoake Tessa L., Enver Tariq, Ratcliffe Peter J., Kranc Kamil R. (2013). Hif-2α is not essential for cell-autonomous hematopoietic stem cell maintenance. Blood.

[25] Velasco-Hernandez Talia, Hyrenius-Wittsten Axel, Rehn Matilda, Bryder David, Cammenga Jörg (2014). HIF-1α can act as a tumor suppressor gene in murine acute myeloid leukemia. Blood.

[26] Velasco-Hernandez T, Tornero D, Cammenga J (2015). Loss of HIF-1α accelerates murine FLT-3ITD-induced myeloproliferative neoplasia. Leukemia.

[27] Wiesener MS, Jurgensen JS, Rosenberger C, Scholze CK, Horstrup JH, Warnecke C (2003). Widespread hypoxia-inducible expression of HIF-2alpha in distinct cell populations of different organs. FASEB J..

[28] Keith B, Johnson RS, Simon MC (2011). HIF1alpha and HIF2alpha: sibling rivalry in hypoxic tumour growth and progression. Nat Rev Cancer..

[29] Forsythe JA, Jiang BH, Iyer NV, Agani F, Leung SW, Koos RD (1996). Activation of vascular endothelial growth factor gene transcription by hypoxia-inducible factor 1. Mol Cell Biol..

[30] Fatrai S, Wierenga AT, Daenen SM, Vellenga E, Schuringa JJ (2011). Identification of HIF2alpha as an important STAT5 target gene in human hematopoietic stem cells. Blood..

[31] Takubo Keiyo, Nagamatsu Go, Kobayashi Chiharu I., Nakamura-Ishizu Ayako, Kobayashi Hiroshi, Ikeda Eiji, Goda Nobuhito, Rahimi Yasmeen, Johnson Randall S., Soga Tomoyoshi, Hirao Atsushi, Suematsu Makoto, Suda Toshio (2013). Regulation of Glycolysis by Pdk Functions as a Metabolic Checkpoint for Cell Cycle Quiescence in Hematopoietic Stem Cells. Cell Stem Cell.

[32] Vannini N, Girotra M, Naveiras O, Nikitin G, Campos V, Giger S, et al. Specification of haematopoietic stem cell fate via modulation of mitochondrial activity. Nat Commun. 2016;713125. 10.1038/ncomms13125. 10.1038/ncomms13125 PMC5064016 27731316

[33] Ito K, Turcotte R, Cui J, Zimmerman SE, Pinho S, Mizoguchi T (2016). Self-renewal of a purified Tie2+ hematopoietic stem cell population relies on mitochondrial clearance. Science..

[34] Ito K, Hirao A, Arai F, Takubo K, Matsuoka S, Miyamoto K (2006). Reactive oxygen species act through p38 MAPK to limit the lifespan of hematopoietic stem cells. Nat Med..

[35] Lagadinou Eleni D., Sach Alexander, Callahan Kevin, Rossi Randall M., Neering Sarah J., Minhajuddin Mohammad, Ashton John M., Pei Shanshan, Grose Valerie, O’Dwyer Kristen M., Liesveld Jane L., Brookes Paul S., Becker Michael W., Jordan Craig T. (2013). BCL-2 Inhibition Targets Oxidative Phosphorylation and Selectively Eradicates Quiescent Human Leukemia Stem Cells. Cell Stem Cell.

[36] Yu Wen-Mei, Liu Xia, Shen Jinhua, Jovanovic Olga, Pohl Elena E., Gerson Stanton L., Finkel Toren, Broxmeyer Hal E., Qu Cheng-Kui (2013). Metabolic Regulation by the Mitochondrial Phosphatase PTPMT1 Is Required for Hematopoietic Stem Cell Differentiation. Cell Stem Cell.

[37] Wilson A, Laurenti E, Trumpp A (2009). Balancing dormant and self-renewing hematopoietic stem cells. Curr Opin Genet Dev..

[38] Essers MA, Trumpp A (2010). Targeting leukemic stem cells by breaking their dormancy. Mol Oncol..

[39] Trumpp A, Essers M, Wilson A (2010). Awakening dormant haematopoietic stem cells. Nat Rev Immunol..

[40] Wierenga AT, Vellenga E, Schuringa JJ (2014). Convergence of hypoxia and TGFbeta pathways on cell cycle regulation in human hematopoietic stem/progenitor cells. PLoS One..

[41] Sontakke P, Koczula KM, Jaques J, Wierenga AT, Brouwers-Vos AZ, Pruis M (2016). Hypoxia-Like Signatures Induced by BCR-ABL Potentially Alter the Glutamine Uptake for Maintaining Oxidative Phosphorylation. PLoS One..

[42] de Boer B, Prick J, Pruis MG, Keane P, Imperato MR, Jaques J (2018). Prospective Isolation and Characterization of Genetically and Functionally Distinct AML Subclones. Cancer Cell..

[43] Sontakke P, Carretta M, Jaques J, Brouwers-Vos A Z, Lubbers-Aalders L, Yuan H, de Bruijn J D, Martens A C M, Vellenga E, Groen R W J, Schuringa J J (2016). Modeling BCR-ABL and MLL-AF9 leukemia in a human bone marrow-like scaffold-based xenograft model. Leukemia.

[44] Yan Q, Bartz S, Mao M, Li L, Kaelin WG (2007). The hypoxia-inducible factor 2alpha N-terminal and C-terminal transactivation domains cooperate to promote renal tumorigenesis in vivo. Mol Cell Biol..

[45] Smirnova N. A., Hushpulian D. M., Speer R. E., Gaisina I. N., Ratan R. R., Gazaryan I. G. (2012). Catalytic mechanism and substrate specificity of HIF prolyl hydroxylases. Biochemistry (Moscow).

[46] van den Boom Vincent, Maat Henny, Geugien Marjan, Rodríguez López Aida, Sotoca Ana M., Jaques Jennifer, Brouwers-Vos Annet Z., Fusetti Fabrizia, Groen Richard W.J., Yuan Huipin, Martens Anton C.M., Stunnenberg Hendrik G., Vellenga Edo, Martens Joost H.A., Schuringa Jan Jacob (2016). Non-canonical PRC1.1 Targets Active Genes Independent of H3K27me3 and Is Essential for Leukemogenesis. Cell Reports.

[47] Schodel J, Oikonomopoulos S, Ragoussis J, Pugh CW, Ratcliffe PJ, Mole DR (2011). High-resolution genome-wide mapping of HIF-binding sites by ChIP-seq. Blood..

[48] Mole DR, Blancher C, Copley RR, Pollard PJ, Gleadle JM, Ragoussis J (2009). Genome-wide association of hypoxia-inducible factor (HIF)-1alpha and HIF-2alpha DNA binding with expression profiling of hypoxia-inducible transcripts. J Biol Chem..

[49] Bagger FO, Sasivarevic D, Sohi SH, Laursen LG, Pundhir S, Sonderby CK (2016). BloodSpot: a database of gene expression profiles and transcriptional programs for healthy and malignant haematopoiesis. Nucleic Acids Res..

[50] McMaster CR (2018). From yeast to humans - roles of the Kennedy pathway for phosphatidylcholine synthesis. FEBS Lett..

[51] Xia X, Lemieux ME, Li W, Carroll JS, Brown M, Liu XS (2009). Integrative analysis of HIF binding and transactivation reveals its role in maintaining histone methylation homeostasis. Proc Natl Acad Sci U S A..

[52] Dang CV (2012). Cancer cell metabolism: there is no ROS for the weary. Cancer Discov..

[53] Kim JW, Tchernyshyov I, Semenza GL, Dang CV (2006). HIF-1-mediated expression of pyruvate dehydrogenase kinase: a metabolic switch required for cellular adaptation to hypoxia. Cell Metab..

[54] Min Irene M., Pietramaggiori Giorgio, Kim Francis S., Passegué Emmanuelle, Stevenson Kristen E., Wagers Amy J. (2008). The Transcription Factor EGR1 Controls Both the Proliferation and Localization of Hematopoietic Stem Cells. Cell Stem Cell.

[55] Peng WX, Xiong EM, Ge L, Wan YY, Zhang CL, Du FY (2016). Egr-1 promotes hypoxia-induced autophagy to enhance chemo-resistance of hepatocellular carcinoma cells. Exp Cell Res..

[56] Ho Theodore T., Warr Matthew R., Adelman Emmalee R., Lansinger Olivia M., Flach Johanna, Verovskaya Evgenia V., Figueroa Maria E., Passegué Emmanuelle (2017). Autophagy maintains the metabolism and function of young and old stem cells. Nature.

[57] Gomez-Puerto MC, Folkerts H, Wierenga AT, Schepers K, Schuringa JJ, Coffer PJ (2016). Autophagy Proteins ATG5 and ATG7 Are Essential for the Maintenance of Human CD34(+) Hematopoietic Stem-Progenitor Cells. Stem Cells..

[58] Mortensen Monika, Soilleux Elizabeth J., Djordjevic Gordana, Tripp Rebecca, Lutteropp Michael, Sadighi-Akha Elham, Stranks Amanda J., Glanville Julie, Knight Samantha, W. Jacobsen Sten-Eirik, Kranc Kamil R., Simon Anna Katharina (2011). The autophagy protein Atg7 is essential for hematopoietic stem cell maintenance. The Journal of Experimental Medicine.

[59] Bellot G, Garcia-Medina R, Gounon P, Chiche J, Roux D, Pouyssegur J (2009). Hypoxia-induced autophagy is mediated through hypoxia-inducible factor induction of BNIP3 and BNIP3L via their BH3 domains. Mol Cell Biol..

[60] Chourasia AH, Macleod KF (2015). Tumor suppressor functions of BNIP3 and mitophagy. Autophagy..

[61] Sena J. A., Wang L., Heasley L. E., Hu C.-J. (2014). Hypoxia Regulates Alternative Splicing of HIF and non-HIF Target Genes. Molecular Cancer Research.

