# Supplementary figures and images for: HIF1/2-exerted control over glycolytic gene expression is not functionally relevant for glycolysis in human leukemic stem/progenitor cells
Source: Cancer Metab. 2019 Dec 27;7:11. doi: 10.1186/s40170-019-0206-y (PMC6935105; doi:10.1186/s40170-019-0206-y)

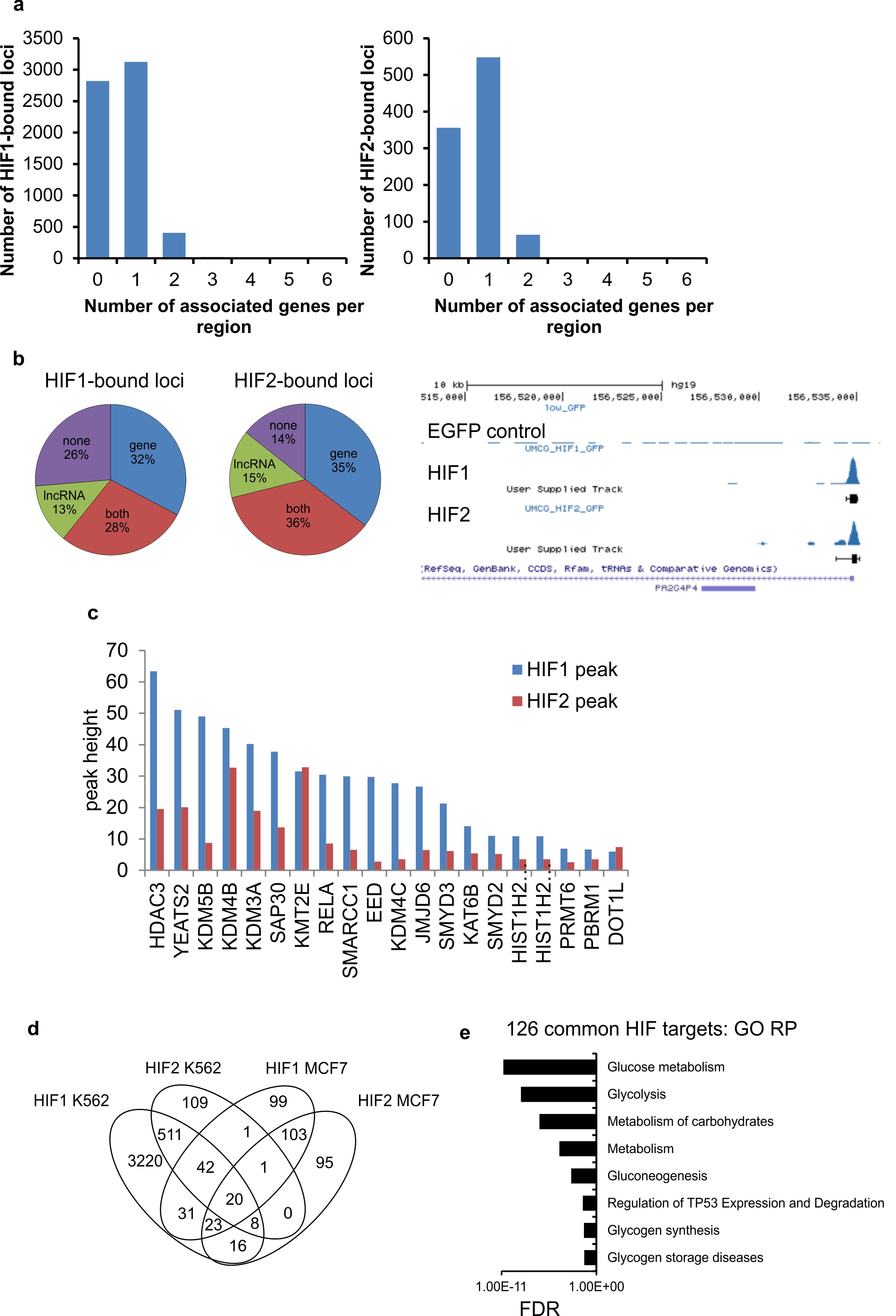

Supplement: Supplementary file 1 — Additional file 1: Figure S1. Identification of HIF1 and HIF2 chromatin binding sites in human leukemic cells. a. About 2/3 of HIF1-bound or HIF2-bound loci localize close to TSSs and 1/3 do not. b. Some HIF1/2 bound loci distal form TSSs potentially regulate lncRNAs. An example screenshot of a lncRNA potentially controlled by HIFs is shown on the right. c. HIF binding to Chromatin modifying enzymes defined by GO RP. d. Overlap in ChIPseq targets in K562 versus MCF7 cells. e. GO reactome pathways in common HIF targets across different cell types. [file 40170_2019_206_MOESM1_ESM.jpg]

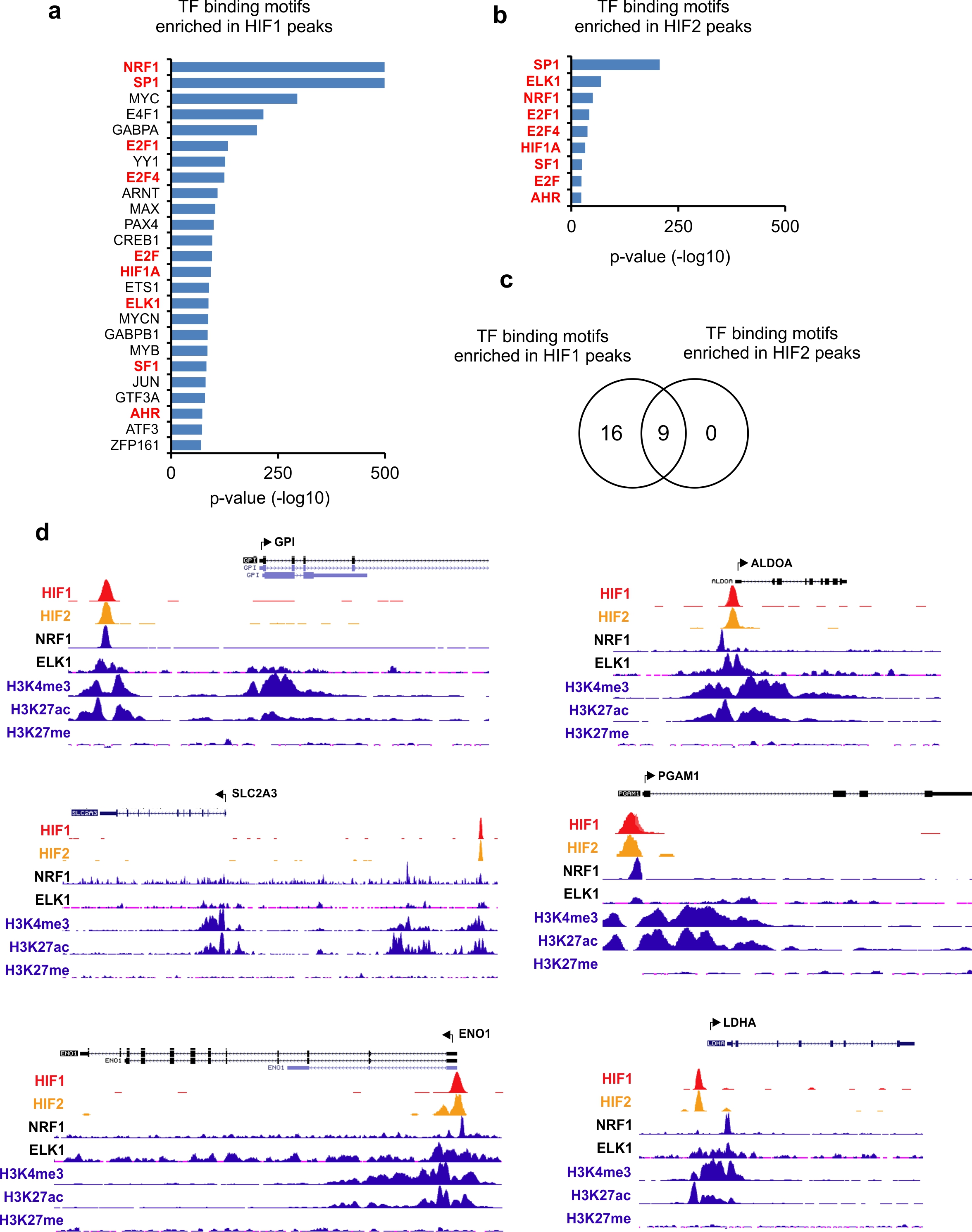

Supplement: Supplementary file 2 — Additional file 2: Figure S2. Identification of co-occuring transcription factor motifs at HIF1 and HIF2-bound loci. A transcription factor (TF) motif screen was performed to identify co-occurring TF motifs at HIF1 (a) and HIF2 (b) loci. The overlap is shown in c. Some representative screens shots are shown in d. NRF1, ELK1, H3K4me3, H3K27ac and H3K27me3 tracks from K562 cells were retrieved from ENCODE. [file 40170_2019_206_MOESM2_ESM.jpg]

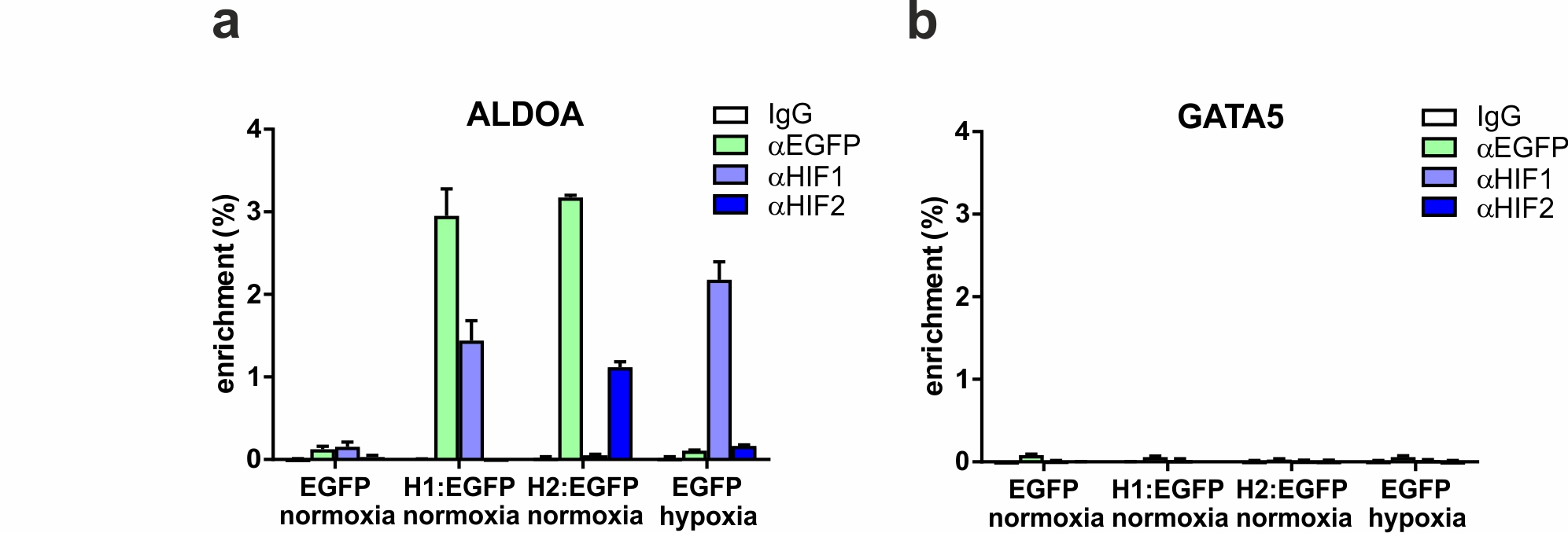

Supplement: Supplementary file 3 — Additional file 3: Figure S3. We overexpressed HIF1 and HIF2 EGFP fusion proteins (in K562 cells) with an empty EGFP expressing vector as control. The cells were sorted for EGFP expression and incubated under normoxia or hypoxia (24 hrs) as indicated. ChIP-QPCR was performed using antibodies against EGFP (recognizing HIF:EGFP fusions), and HIF1 and HIF2 (recognizing HIF:EGFP fusions as well as endogenous HIF). b. No HIF was present on the GATA5 locus to which HIFs do not bind. [file 40170_2019_206_MOESM3_ESM.jpg]

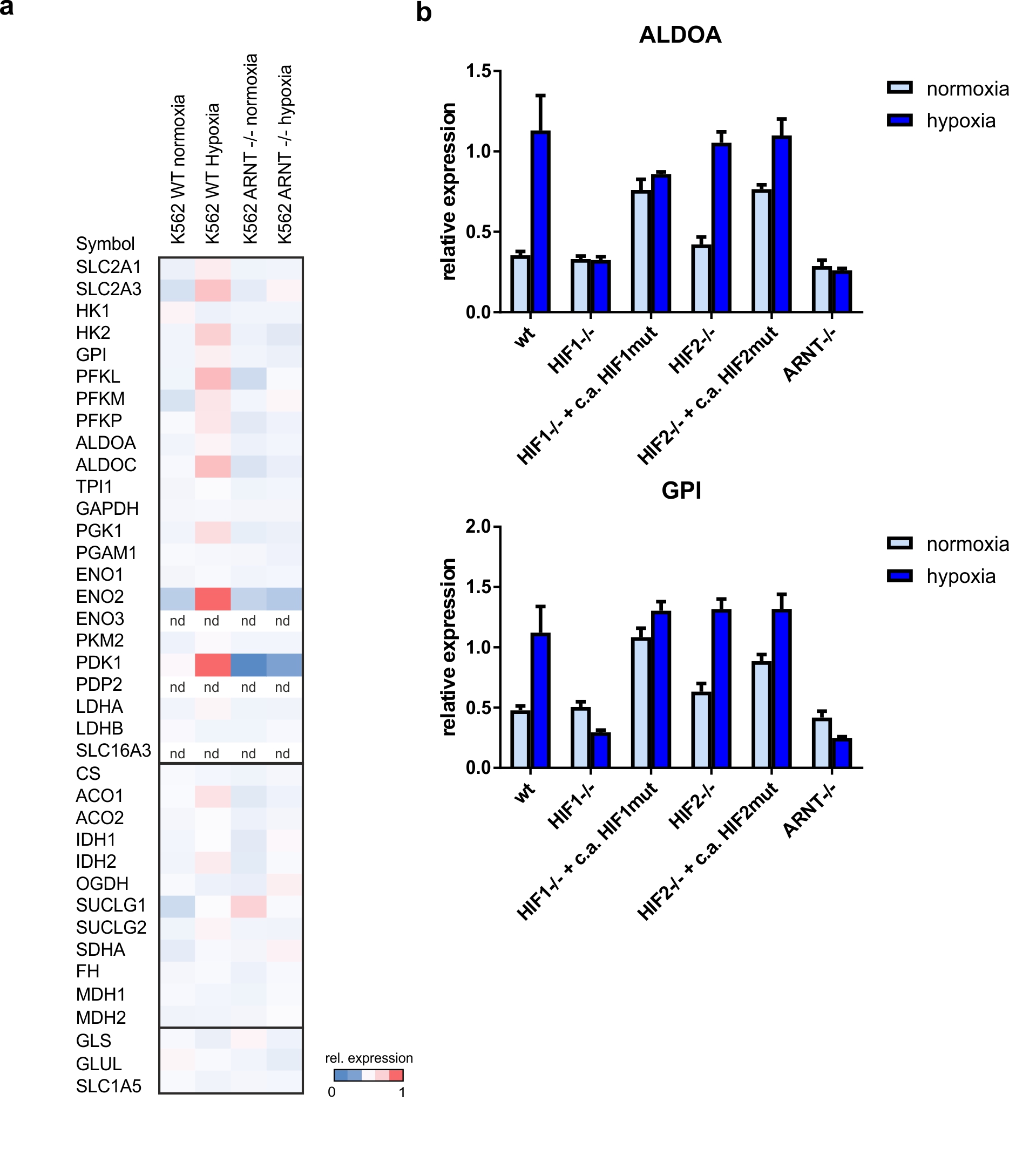

Supplement: Supplementary file 4 — Additional file 4: Figure S4. a. Quantitative proteome data of wt or ARNT-/- K562 cells grown under hypoxia (24 hrs) or normoxia and protein expression levels are shown for glycolysis, TCA and glutaminolysis-related proteins. The K562 wt normoxia/hypoxia data are identical to those used for Figure 3b (where fold changes are shown rather than relative protein expression levels), but are again shown here to compare with ARNT-/- cells. b, Rescue experiment by re-introducing the oxygen-insensitive HIF1 and HIF2 mutants in the HIF1-/- or HIF2-/- K562 cells, respectively. Q-RT-PCRs were performed indicating that glycolysis-related genes can be re-expressed upon overexpression of HIF mutants. [file 40170_2019_206_MOESM4_ESM.jpg]

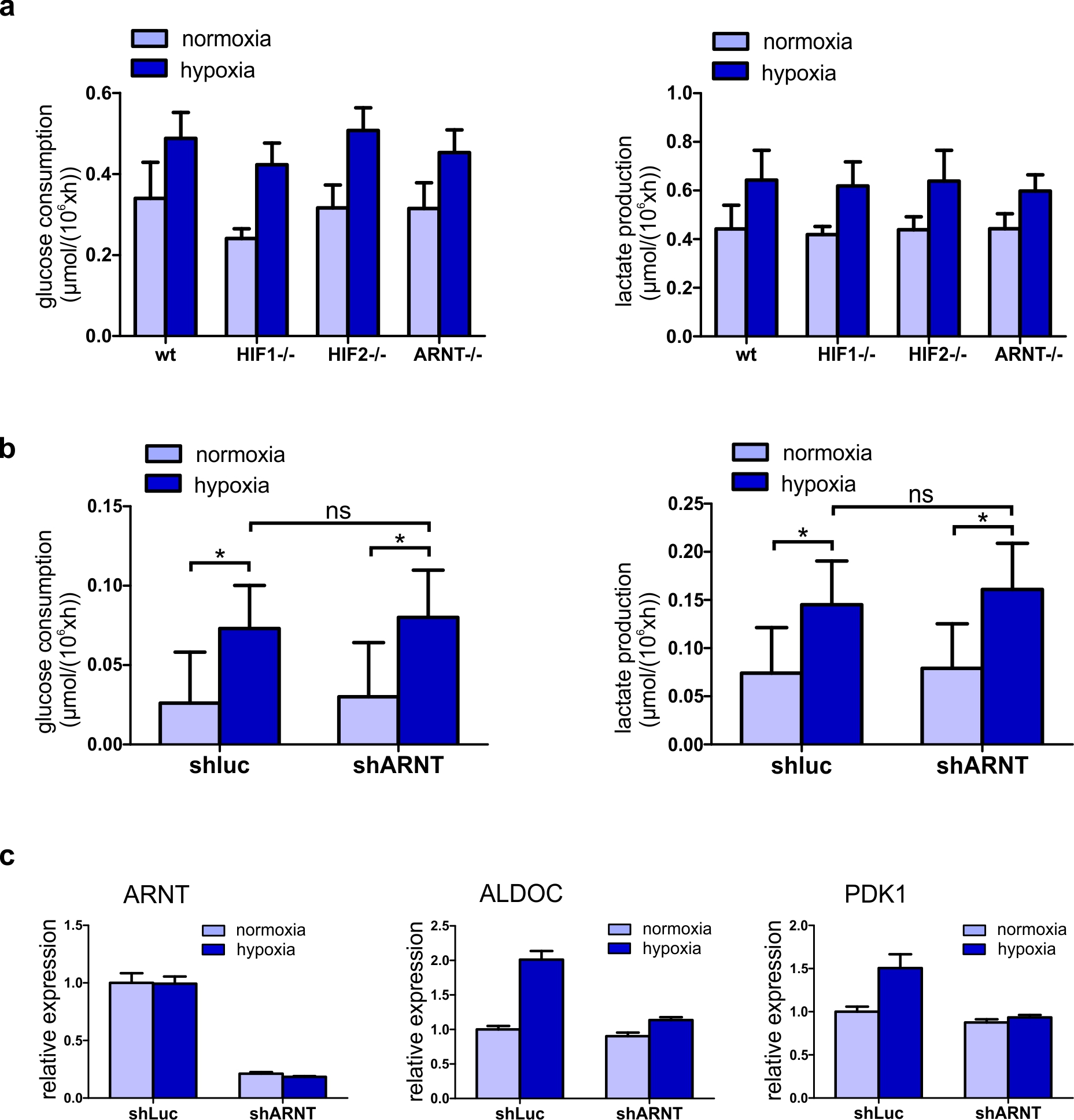

Supplement: Supplementary file 5 — Additional file 5: Figure S5. Glucose consumption and lactate production in CB CD34+ and K562 cells. a. Glucose consumption (left panel) and lactate production (right panel) of K562 cells, grown for 10 days under hypoxia (1% O2). *: P<0.05, n.s.: Non-significant. b. Glucose consumption (left panel) and lactate production (right panel) of cordblood. CD34+ cells after 24 hour hypoxia (1% O2), with knockdown of ARNT. c. Knockdown efficiency of ARNT (left panel) and expression of target genes (middle and right panel) in cordblood CD34+ cells. *: P<0.05, n.s.: Non-significant. [file 40170_2019_206_MOESM5_ESM.jpg]

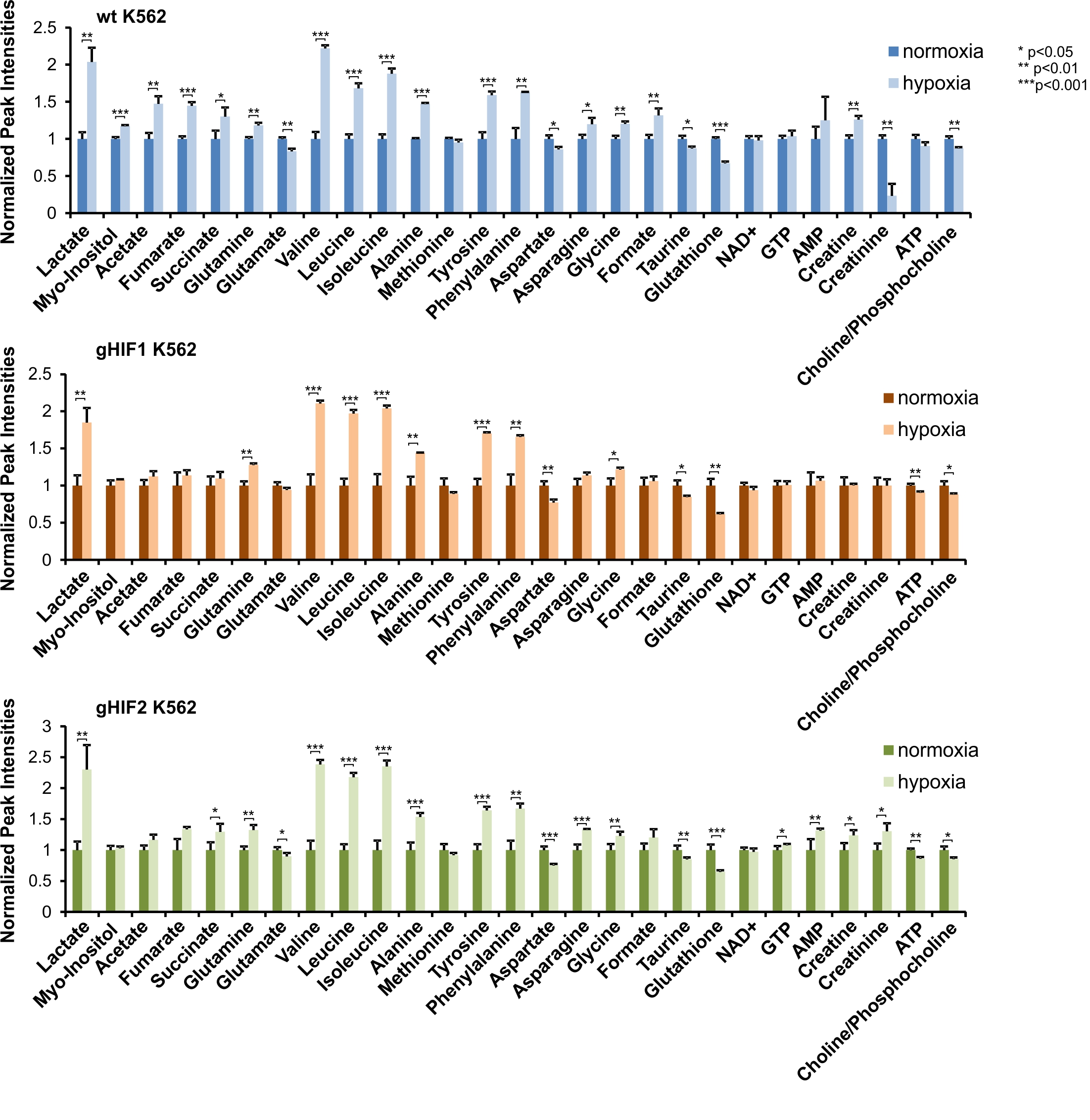

Supplement: Supplementary file 6 — Additional file 6: Figure S6. 1D-NMR extract metabolite intensities from K562 HIF1 and HIF2 knockout cells grown under hypoxia or normoxia for 24 hr. The K562 wt data is identical to that depicted in Fig. 6b but was added here again for reference. [file 40170_2019_206_MOESM6_ESM.jpg]
