# Supplementary material for: HIF1/2-exerted control over glycolytic gene expression is not functionally relevant for glycolysis in human leukemic stem/progenitor cells
Source: Cancer Metab. 2019 Dec 27;7:11. doi: 10.1186/s40170-019-0206-y (PMC6935105; doi:10.1186/s40170-019-0206-y)
Supplement: Supplementary file 7 — Additional file 7. Supplemental Methods. [file 40170_2019_206_MOESM7_ESM.pdf]

**Supplemental methods**

# HIF1/2-exerted control over glycolytic gene expression is not functionally relevant for glycolysis in human leukemic stem/progenitor cells

Albertus T.J. Wierenga[1,2], Alan Cunningham[1*], Ayşegül Erdem[1*], Nuria Vilaplana Lopera[3*], Annet Z. Brouwers-Vos[1], Maurien Pruis[1], André B. Mulder[2], Ulrich L. Günther[3], Joost H.A. Martens[4], Edo Vellenga[1] and Jan Jacob Schuringa[1#].

[1]Department of Experimental Hematology, [2]Department of Laboratory Medicine, University Medical Center Groningen, University of Groningen, Hanzeplein 1, 9700 RB, Groningen, The Netherlands. [3]Institute of Cancer and Genomic Sciences, University of Birmingham, University of Birmingham, UK. [4]Department of Molecular Biology, Radboud Institute for Molecular Life Sciences (RIMLS), Nijmegen, the Netherlands.

**Cell culture and lentiviral transductions.**

Neonatal cord blood (CB) was obtained from healthy full-term pregnancies from the Obstetrics departments of the University Medical Center and Martini Hospital in Groningen, The Netherlands, after informed consent. The protocol was approved by the Medical Ethical Committee of the UMCG. Donors are informed about procedures and studies performed with CB by an information sheet that is read and signed by the donor, in line with regulations of the Medical Ethical Committee of the UMCG. CB $CD34^+$ cells were isolated with the use of a hematopoietic progenitor isolation kit from Miltenyi Biotech according to the manufacturer's instructions. OCI-AML3 (ACC-582), K562 (ACC-10) and HL60 (ACC-3) cells were purchased from DSMZ (Braunschweig, Germany) and cultured in RPMI 1640 (Lonza, Verviers, Belgium) supplemented with 10% FBS, 1 mmol/L L-glutamine, and 100 U/mL penicillin/streptomycin (Life Technologies, Bleiswijk, the Netherlands). Lentiviral vectors expressing constitutively active HIF1(P402A,P564A) and HIF2(P405A,P531A) were constructed by cloning the HA-tagged HIF cDNAs, from pCDNA3 vectors obtained from Addgene (Addgene plasmid numbers 18955 and 18956) into the pRRL-SFFV-iresEGFP vector. For ChIP experiments, the cDNAs for the constitutively active HIFs were cloned as EGFP fusions in the pRRL-SFFV vector to be able to perform pull down experiments using an anti-EGFP antibody. A lentiviral vector expressing a short hairpin in a MirE background was made by swapping the GFP:MirE fusion cassette from the dox-inducible pRRL-GEPIR (kindly provided by J. Zuber, Research Institute of molecular Pathology, Vienna, Germany) into the pRRL-SFFV backbone to create a constitutively MirE-shRNA expressing vector. A shRNA against ARNT was cloned into the vector (ARNT 464 in the Supplementary table by Fellman et al [1]), as well as a shRNA targeting luciferase (as a

non-relevant control vector). Lentiviral transductions were essentially performed as described elsewhere [2-4]. In short, isolated $CD34^+$ cells were cultured for 48 hours in serum free medium (HPGM, Lonza, Breda, the Netherlands) containing 100 ng/ml each of SCF, TPO and FLT-3 ligand. Cells were subsequently transduced with lentiviral particles in one to two consecutive rounds of 12 hours in the presence of 4 µg/ml polybrene. For cell lines, 200,000 cells were transduced in 12 well plates in 1 ml of virus containing medium in the presence of 8 µg/ml polybrene. After transduction, cells were sorted based on EGFP expression using a MoFlo XDP cell sorter (Beckman Coulter).

**Generation of CRISPR/Cas9 knockout lines**

**Plasmid construction:**

A lentiviral vector expressing the guide RNA molecules (pLsgRNA-mBlueberry2) was constructed by modification of the pLKO-puro (Addgene ID: 8453). First, the cDNA encoding the puromycin resistance was replaced by a cDNA encoding mBlueberry2 (kind gift of R.E. Campbell, dept. of Pharmacology and Chemistry, Howard Hughes Medical Institute, University of California, CA, USA), followed by removal of the BbsI site in the backbone of the vector. Second, a synthetic DNA stretch as described in [5] was inserted downstream of the U6 promoter. Expression of the guide RNA from the U6 promoter then resulted in expression of the guide RNA sequence fused to an engineered gRNA scaffold with an extended Cas9 binding hairpin. Oligonucleotides coding for the desired gRNA sequences were then cloned into the BbsI sites of the vector, using the method described in [6]. Clones were sanger sequenced to verify the correct sequence. The full sequence of the pLsgRNA-mBlueberry2 is available upon

request. A vector expressing Cas9 fused to EGFP (pMJ920) was obtained from Addgene (Addgene ID: 42234).

**Guide RNA selection:**

The online platform Benchling (www.benchling.com) was used to design guide RNA sequences for HIF1, HIF2 and ARNT. For all genes, two different gRNAs were selected based on high on-target and off-target scores. gRNA sequences and the highest scoring off-targets are listed in table 1.

**Procedure:**

K562 cells (1 x$10^7$) were transiently transfected by electroporation using a custom made square wave pulser with a pulse height of 140 V and a pulse length of 80 ms in a 2 mm gap width cuvette in 200 µl RPMI 1640 medium, with 5 µg pMJ920 and 5 µg pLsgRNA-mBlueberry2. After electroporation, cells were immediately resuspended in RPMI1640 medium containing 10% FCS. 48 hours later, EGFP and mBlueberry2 double positive cells were single cell sorted in 96 well plates in 150 µl of growth medium using a MoFlo XDP cell sorter (Beckman Coulter). Plates were incubated for 12 days after which positive wells were harvested and replated in 24 well plates in which they were kept during the analysis of the clones. Genomic DNA of the clones was extracted and the gRNA targeted region, as well as the highest scoring off-target (as predicted by the Benchling software) was PCR amplified and sequenced by Sanger sequencing (PCR primers are listed in table 2). Sequencing tracks were analyzed using the online available TIDE analysis tool (www.tide.nki.nl). All off-targets were found to be unaffected. Clones with lesions resulting in premature stop codons on both alleles were

expanded and two different clones of each guide RNA were frozen. For experiments, clones were either used individually or four clones (with the same knockout) were pooled.

**Table 1: guide RNA targets and highest scoring off-targets**

| gene | guide | exon | Target sequence | Highest off-target (score) |
|---|---|---|---|---|
| HIF1 | 1 | 5 | AAGTGTACCCTAACTAGCCG | HIF3A (0.1) |
| | 2 | 5 | TGAACATAAAGTCTGCAACA | C12orf66 (0.6) |
| HIF2 | 1 | 2 | AGATGGGAGCTCACACTGTG | AC099850.1 (0.6) |
| | 2 | 2 | GATTGCCAGTCGCATGATGG | RP5-1065J22.8 (0.6) |
| ARNT | 1 | 6 | CGAGCCAGGGCACTACAGGT | LENG1 (0.6) |
| | 2 | 6 | CAAGCTAACCATCTTACGCA | LINC00327 (0.3) |

**Table 2: primers used for sequencing of CRISPR/Cas9 targets**

| target | forward | reverse |
|---|---|---|
| HIF1 | CTCTTGGGTGTGCTGGAGTGAG | GTGGCTGGCAAAGCCAACTG |
| HIF2 | GAGCCCAGATCAGTCTAGTAAG | AAAGGTCACTGCTGGAAGAG |
| ARNT | TCTTCAGCAGCACACTTG | CCTCTGTGATTCCACTGTTC |
| HIF3 | CCTGTGGAACACCAGACTAAG | CCCATAGGAACCCATCCTAAG |
| C12orf66 | CTTCCAAGAAGGAGGGAAAC | CCGGAACCTCACTTTACC |
| AC099850.1 | AGCCACACCCGTAGTTAAG | GCACTCTAGGTCGGTTAAG |
| RP5-1065J22.8 | AGGCCAGCTTCCAGAGGATG | CCTGGGCAACAAGAGCGGAAAC |
| LENG1 | CCGGGAGGTTTTCCTCTCA | GGGGCTGCTAAGTGTGCTTTC |
| LINC00327 | CGGAAGAATTCCTAGCCCTTAC | ACCCGCAATTTGCAAGACAG |

**Table 3: genetic lesions of CRISPR/Cas9 clonal K562 cell lines**

| Gene (transcript #) | Guide RNA | Clone # | First allele | | Second allele | |
|---|---|---|---|---|---|---|
| | | | c.Nomen | p.Nomen | c.Nomen | p.Nomen |
| HIF1 (NM_181054.2) | 1 | H1_1_1 | c.530del | p.S177Tfs*6 | c.532dup | p.R178Pfs*25 |
| | | H1_1_7 | c.530del | p.S177Tfs*6 | c.527_530del | p.S177Qfs*5 |
| | 2 | H1_2_1 | c.562dup | p.T188Nfs*15 | homozygous | |
| | | H1_2_5 | c.562dup | p.T188Nfs*15 | homozygous | |
| HIF2 (NM_001430.4) | 1 | H2_1_3 | c.136dup | p.S46Kfs*31 | c.124_136del | p.P42Vfs*2 |
| | | H2_1_6 | c.136dup | p.S46Kfs*31 | c.124_136del | p.P42Vfs*2 |
| | 2 | H2_2_3 | c.166del | p.I56Sfs*37 | homozygous | |
| | | H2_2_4 | c.166del | p.I56Sfs*37 | c.166dup | p.I56Nfs*21 |
| ARNT (NM_001668.3) | 1 | A_1_2 | c.355dup | p.C119Lfs*2 | homozygous | |
| | | A_1_3 | c.355dup | p.C119Lfs*2 | c.354_355insA | p.C119Mfs*2 |
| | 2 | A_2_13 | c.397dup | p.R133Pfs*19 | homozygous | |
| | | A_2_16 | c.397dup | p.R133Pfs*19 | homozygous | |

**Chromatin immunoprecipitation**

For ChIP, cells were either incubated in an hypoxic incubator (1% $O_2$, 5% $CO_2$) for 24 hours or left in normoxia, washed with PBS and fixed with 1% formaldehyde in PBS, after which the formaldehyde was quenched by adding 0,1 volume of 1,25 M glycin. Cells were centrifuged, resuspended in 200 µl SDS lysis buffer (50 mM Tris pH 8,1, 100mM $NaCl_2$, 5 mM EDTA, 10 % SDS) and stored in -80°C. After thawing, the cell pellet (containing nuclei) was recoverd by centrifugation and resuspended in IP buffer (SDS lysis buffer, diluted with Triton dilution buffer (100 mM Tris pH8,6, 100 mM $NaCl_2$, 5 mM EDTA, 5% Triton X-100) at a 1 to 0.5 ratio respectively), and sonicated using a Bioruptor (Diagenode SA, Seraing, Belgium) for 15 minutes at setting "high". Subsequently, the chromatin was further sheared using two 30-second pulses of a tip sonicator (Soniprep 150, MSE), diluted to an appropriate volume depending on the number of ChIP reactions with IP buffer and centrifuged for 30 minutes at 20.000 rcf to remove cellular debris. The supernatant was precleared with 30 µl Protein G magnetic beads (Dynabeads, Thermofisher), for 30 minutes at 4°C after which the cleared supernatant was recovered.

ChIP reactions were performed in 96-well deep well plates, with 150 µl chromatin extract. 3 µg of antiboby was added per ChIP reaction and the plate was rocked overnight on an orbital shaker. Next day, 25 µl Protein G beads was added and shaken for another 2 hours. Beads were captured in the plate by the use of a magnetic plate and supernatant was aspirated. Beads were washed three times in Mixed Micelle buffer (20 mM Tris pH 8.1, 150 mM $NaCl_2$, 5 mM EDTA, 5% sucrose, 1% Triton X-100, 0.2% SDS), two times in Buffer 500 (50mM HEPES pH 7.5, 500mM $NaCL_2$, 0,1% deoxycholic

acid, 1 mM EDTA), two times in $LiCl_2$ wash buffer (10 mM Tris pH8.0, 0.5% deoxycholic acid, 1 mM EDTA, 250 mM $LiCl_2$, 0,5% NP-40) and once in TE. The precipitated complexes were eluted from the beads by shaking in 100 µl elution buffer (1% SDS, 0.1M $Na_2HCO_3$) at 65 °C for 30 minutes. The beads were captured and the supernatant was recovered. Proteins complexes were reverse crosslinked at 65°C overnight. Subsequently, the supernatant was treated with RNAse A for 30 minutes at 37 °C and digested with Proteinase K for 2 hours (0.2 µg/µl) at 55°C. DNA was purified either by Qiagen PCR Purification Kit (Qiagen) or AMPure XP beads (Beckman Coulter) according to the manufacturer's instructions. Purified DNA was used either for NGS or for qPCR analysis.

**ChIP-seq**

Sequencing samples were prepared according to the manufacturer's protocol (Illumina). End repair was performed using the precipitated DNA using Klenow and T4 PNK. A 3' protruding A base was generated using Taq polymerase and adapters were ligated. The DNA was loaded on gel and a band corresponding to ~300 bp (ChIP fragment + adapters) was excised. The DNA was isolated, amplified by PCR and used for cluster generation on the Illumina NextSeq500 genome analyzer. The 50 bp tags were mapped to the human genome HG19 using BWA [7]. For processing and manipulation of SAM/BAM files, SAMtools was used [8]. For each base pair in the genome the number of overlapping sequence reads was determined and averaged over a 10 bp window and visualized in the UCSC genome browser [9]. ChIP-seq data is deposited at GEO under GSE123461.

### Detection of enriched regions

Peak calling algorithm MACS was used to detect the binding sites at a q-value cut off for peak detection of 0.01. ChIP-seq tracks were visualized using UCSC genome browser [9]. Identification of genes associated to detected peaks was performed using GREAT [10].

### Tag counting

Tags within a given region were counted and adjusted to represent the number of tags within a 1 kb region. Subsequently the percentage of these tags as a measure of the total number of sequenced tags of the sample was calculated.

### Generation of profiles and heatmaps

Heatmaps and bandplot profiles were generated using fluff [11].

### Chip-qPCR

qPCR analysis of the ChIP DNA was performed using SsoAdvanced Universal SYBR Green Supermix (Biorad) in a CFX384 Touch thermal cycler. Data was analysed using CFX manager software (Biorad). Input samples (taken from chromatin before immunoprecipitation) were analysed in parallel and used to calculate the enrichment of the precipitated samples (percentage of input). Primer sequences of the used primers are listed in table 4.

**Table 4: Primers used for ChIP-qPCR**

| Promoter region | forward | reverse |
|---|---|---|
| SLC2A1 | ATGGCCGGGGTCCTATAAACG | ACGCTCGCTGTTGCTACCT |
| SLC2A3 | GGAGGCTGAGACAGCAGATAC | TCGCCACCCAAAGACTCAC |

| | | |
|---|---|---|
| ALDOA | CCAACATTCTGGCTGAGTC | TCCCTTCTGCTCCTTTCC |
| PDK1 | TCCATACCGCCTCGCCTCTTAG | TACACGTCGGGTGATGGGACTG |
| PKM | TGCTGGCATGAGGAAAGAGG | TGTGCTTGTCTGCACGTAGG |
| PFKFB3 | TTTCCGCCGCGGTGTAGGTTTC | TGAGGCTGGGCTGTGATTGGAG |
| GPI | GGCCAACACCAGGGACAAAC | TGCTTGGAGAGGCCACCTAC |

**mRNA Analysis**
**Table 5: Primers used for RT-qPCR**

| target | forward | reverse |
|---|---|---|
| PDK1 | CAGGACAGCCAATACAAGTG | GTTGGCATGGTGTTCCATAG |
| PDK3 | CCAGAGCTGGAAGTTGAAG | GTGAGGGCACATAAACCAC |
| SLC2A1 | TTGTGGGCATGTGCTTCCAG | ATCGAAGGTCCGGCCTTTAG |
| SLC2A3 | CGTCGGACTCTTCGTCAAC | CACCAGTGACAGCCAACAG |
| BNIP3 | AGCTCACAGTCTGAGGAAGATG | CGCTTCGGGTGTTTAAAGAGG |
| ALDOA | TTGTGGGCATCAAGGTAG | TAGTCTCGCCATTTGTCC |
| GPI | AAATCGCCCAACCAACTC | ATGATGCCCTGAACGAAG |
| PKM | GCCTGTCATCTGTGCTACTC | CTCCATCCAGGACTGCATTG |
| ARNT | ACCAGCAGCTTCTAAGAGAC | TCCAGAGCCATTCTTGGTTC |
| ALDOC | ATCGTCGTGGGCATCAAGG | TTGGGCACAGCGTTCTGAG |

**Long noncoding RNA – peak association**

The potential association between HIF1 and HIF2 peaks and promoter regions of Long noncoding (Lnc) RNA was calculated using Galaxy Tools (www.usegalaxy.org) [12]. First, the “high confidence set”, version 5.0, hg19 of LncRNAs was downloaded from LNCipedia.org as .bed file and imported in Galaxy. Using “get flanks”, the regions spanning 5000 bp upstream and 1000 bp downstream of the transcription start site of the LncRNAs were retrieved and merged into a -5000/+1000 .bed file. Next, using the “coverage” tool in Galaxy, the overlap between the .bed file of the peaks of HIF1 or HIF2 and the -5000/+1000 .bed file was calculated. An association was considered to be present if there was overlap of at least 1 bp. The genes associated with the overlapping regions were retrieved using GREAT (www.great.stanford.edu) by uploading the .bed file of the associated peaks.

**Geneset enrichment analysis**

Geneset enrichment analysis (GSEA) was performed using software from the Broad Institute (http://software.broadinstitute.org/gsea/index.jsp). Custom genesets were made by taking the highest 200 HIF1 and HIF2 peaks from the ChIP-seq data (this paper), based upon the tag counts of the peaks. Using GREAT (www.great.stanford.edu), the gene names associated with the peaks were retrieved and packed into a .grp file for HIF1 and HIF2 respectively. The ranked lists of gene expression changes after over expression of HIF1, HIF2 or by incubation in hypoxia were made using Genespring software (Agilent Technologies), whereby duplicate gene names were removed using an in-house developed application.

**Proteome studies**

Total cell lysates were prepared from K562 cells (wild type and ARNT knockout lines, grown under normoxia or for 24 hr under hypoxia, both in duplicate) by lysing $4x10^6$ cells in 300 µl RIPA buffer on ice for 15 minutes and subsequent centrifugation. Protein concentration in the cleared supernatant was measured with a BCA assay (Thermo Scientific) and 30 µg protein per sample was separated on 4-15% Mini-Protean TGX gels (BioRAD), stained with Coomassie blue G250, followed by destaining with ultrapure water, and cut into 8 slices for in-gel trypsin digestion. Coomassie-stained slices were further cut into small pieces and destained using 70% 50 mM $NH_4HCO_3$ and 30% acetonitrile. Reduction was performed using 10 mM DTT dissolved in 50 mM $NH_4HCO_3$ for 30 min at 55°C. Next the samples were alkylated using 55 mM iodoacetamide in 50 mM $NH_4HCO_3$ for 30 min at room temperature and protected from

light. Subsequently, samples were washed for 10 min with 50 mM $NH_4HCO_3$ and for 30 min with 100% acetonitrile. Remaining fluid was removed and gel pieces were dried for 15 min. at 55°C. Tryptic digest was performed by addition of sequencing-grade modified trypsin (10 ng/µl in 50 mM $NH_4HCO_3$) and overnight incubation at 37°C. Peptides were extracted using 5% formic acid followed by a second elution with 5% formic acid in 75% acetonitrile. Samples were dried in a SpeedVac centrifuge and dissolved in 5% formic acid. For LC-MS/MS analyses, online chromatography of peptides was performed with an Ultimate 3000 nano-HPLC system (Thermo Fisher Scientific) coupled online to a Q-Exactive-Plus mass spectrometer with a NanoFlex source (Thermo Fisher Scientific) equipped with a stainless steel emitter. Tryptic digests were loaded onto a 5 mm × 300 µm i.d. trapping micro column packed with PepMAP100 5 µm particles (Dionex) in 0.1% FA at the flow rate of 20 µL/min. After loading and washing for 3 min, peptides were forward-flush eluted onto a 50 cm × 75 µm i.d. nanocolumn, packed with Acclaim C18 PepMAP100 2 µm particles (Dionex). The following mobile phase gradient was delivered at the flow rate of 300 nL/min: 3–50% of solvent B in 90 min; 50–80% B in 1 min; 80% B during 9 min, and back to 3% B in 1 min and held at 3% B for 19 min. Solvent A was 100:0 H2O/acetonitrile (v/v) with 0.1% formic acid and solvent B was 0:100 H2O/acetonitrile (v/v) with 0.1% formic acid. MS data were acquired using a data-dependent top-10 method dynamically choosing the most abundant not-yet-sequenced precursor ions from the survey scans (300–1650 Th) with a dynamic exclusion of 20 s. Sequencing was performed via higher energy collisional dissociation fragmentation with a target value of 1e5 ions determined with predictive automatic gain control. Isolation of precursors was performed with a window of 1.8 Da. Survey scans were acquired at a resolution of 70,000 at m/z 200. Resolution for HCD spectra was set to 17,500 at m/z

200 with a maximum ion injection time of 50 ms. Normalized collision energy was set at 28. Furthermore, the S-lens RF level was set at 60 and the capillary temperature was set at 250degr. C. Precursor ions with single, unassigned, or six and higher charge states were excluded from fragmentation selection. Raw mass spectrometry data were analyzed using MaxQuant version, 1.5.2.8 [13], using default settings and LFQ/iBAQ enabled, and searched against the Human Uniprot/Swissprot database (downloaded June 26, 2016, 20197 entries). The data was further processed using Perseus software, version 1.5.8.5 [14].

**Glucose consumption and lactate production assays**

K562 cells were grown for 24 hrs in RPMI growth medium with 10% FCS and 1% Penicillin and streptomycin at a density $500x10^3$/ml. Aliquots were removed for both cell counting and for medium collection. Medium samples were centrifuged and the whole supernatant collected and stored at -20˚C until further analysis. Medium samples were defrosted prior to analysis and diluted 1:10 in $dH_20$ and 25 µl or 20 µl pipetted into 96 well flat bottom plates for glucose or lactate measurements respectively. Glucose and lactate standards ranging in concentration from 0 mM to 1.4 mM were prepared in $dH_20$ and 25 µl or 20 µl respectively was added to a 96 well plates. To determine glucose concentration, a enzymatic reagent mix consisting of 75 µl 100 mM PIPES buffer, 2.5 µl 40 mM NADP, 2 µl 10 mM ATP, 1 µl 500 mM MgSO4, 0.15 µl hexokinase, 0.15 µl glucose-6-phosphate-dehyrogenase and 44.3 µl $H_20$ per well was prepared and 125 µl added to both medium and glucose standard wells. The plate was incubated for 10 mins at 37˚C and absorbance measured by spectrometry at a wavelength of 340 nm. Lactate levels were determined by preparing a reagent mix of 114.5 µl assay buffer (0.5M

glycine/0.4M hydrazine pH 9), 15 µl 25 mM NAD and 0.5 µl lactate dehydrogenase per reaction well. 130 µl of reaction mix was added to each medium and standard well and incubated for 30 minutes at 37˚C. Absorbance was measured at a wavelength of 340 nM. Glucose and lactate levels were determined from their respective standard curves. The rate of consumption and production of both metabolites was calculated based on i; the difference in the level of glucose or lactate in the medium between the start and the end of the experiment, ii) the initially seeded number of cells and the number of cells present at the end of the experiment, and iii) the amount of time that cells were cultured, which yielded the amount of µmol glucose consumed or lactate produced per $10^6$ cells per hour.

**Statistical analyses**

All statistical analyses was performed using the student t test (unpaired, two-tailed) and were expressed as means ± SEM for all other comparisons. Differences were considered statistically significant at $p \leq 0.05$.

**Extraction of polar metabolites and NMR spectroscopy**

K562 CRISPR-Cas9 knockout cells were used for NMR analyses. For *in vitro* studies, $10 \times 10^6$ cells (+/- 24 hours of hypoxia) from each flask were isolated and centrifuged at 400 rcf for 5 minutes. The supernatant was discarded and cells resuspended in 1ml ice cold PBS and centrifuged for 30 seconds at 10000 rcf at 4˚C. The supernatant was discarded and the pellet rapidly resuspended and quenched in 400 µl MeOH (HPLC grade) pre-chilled on dry ice. Eppendorfs were placed on dry ice and further stored at -80˚C or immediately extracted. For the extractions, quenched samples were transferred

to glass vials and 325 µl Milli-Q water and 400 µl chloroform (HPLC grade), pre-chilled on wet ice, was added. Samples were vortexed for 30 seconds prior to resting for 10 minutes and subsequent centrifugation for 15 minutes at 4000rpm. Samples were further rested for 10 minutes and 400 µl from the polar phase removed using a glass Hamilton syringe. For the *in vivo* studies, viable tumor cells were isolated by density gradient centrifugation using Lymphoprep (Alere technologies, Oslo, Norway) and polar metabolites. $7.25 \times 10^6$ tumor cells per sample were then extracted as above. The polar phases from all samples were evaporated using a SpeedVac concentrator and stored at -80˚C until further analysis. Medium samples were collected at 18, 21 and 24 hrs following culture under normoxia or hypoxia and cells were counted. The samples were centrifuged and the complete supernatant removed to a separate Eppendorf which was frozen at -80˚C until further analysis. For the NMR analysis, dried cell extracts were re-suspended in 50 µl of 100 mM sodium phosphate buffer containing 0.5 mM TMSP ((3-trimethylsilyl)propionic-(2,2,3,3-d4)-acid sodium salt) in 90% H2O/10% D2O, at pH 7.0. Media samples were prepared using concentrated buffer containing TMSP to reach a final phosphate buffer concentration of 100 mM at a pH of 7 and a TMSP concentration of 0.5 mM to a final volume of 180 µl. Cell extract samples were vortexed, sonicated and centrifuged briefly, before being transferred into a 1.7 mm NMR tube. Media samples were transferred to 3 mm NMR tubes using a glass pipet. All NMR data was acquired at Bruker 600 MHz spectrometers equipped with Avance-III consoles. For cell extracts a TCI 1.7 mm z-PFG cryogenic probe was used, for media samples in 3 mm NMR tubes we used a 5 mm TCI z-PFG cryogenic probe; both probes were equipped with an automated tuning and matching unit. Spectra were acquired at 300K using a 1D 1H-

NOESY pulse sequence with presaturation water suppression with 64 scans for media samples and 128 scans for cell extracts. The spectral width of all spectra was 12ppm with 32,768 data points. Spectra were processed using the MATLAB-based MetaboLab software [15]. 0.5 Hz line broadening and zero-filling up to 131,072 data points were applied before Fourier transformation. The chemical shift was calibrated by referencing the TMSP signal to 0 ppm. Spectra were manually phased corrected, a spline baseline correction was applied and spectra were scaled to probabilistic quotient normalization (PQN). Chenomx 7.0 software (Chenomx Inc.) was used to assign the metabolites present in the acquired spectra. Peak intensities were obtained directly from the spectra. The intracellular metabolite measurements are presented as relative peak intensities. For extracellular analysis the peak intensities were corrected for the initial intensities (blank medium sample) and subsequently corrected for the cell number and incubation time by calculating the rate of consumption or production. For this, the area under the curve (AUC) at a certain time point was calculated using the cell counts, the initial seeding density and the incubation time. The peak intensities at different time points were then divided by the respective AUC, resulting in a rate of consumption or production. The rates at 18, 21 and 24 hour incubation time were averaged to get an average rate per metabolite (expressed as intensity per cell x hour).

**mRNA analysis**

Total RNA was isolated using the RNeasy kit from Qiagen (Qiagen, Venlo, The Netherlands) according to the manufacturer's recommendations. For real-time RT-PCR, cDNA was prepared using the iScript cDNA synthesis kit (Bio-Rad, Veenendaal, the Netherlands) and cycling was performed using SsoAdvanced SYBR green Supermix

(Bio-Rad) in a CFX394 Touch thermocycler (Bio-Rad), and quantified using CFX software (Bio-Rad). Ribosomal Protein Like (RPL) 27 expression levels were used to normalize between samples and to calculate relative expression levels. Primers are listed in Supplementary Methods, Table 5. Genome-wide expression analysis was performed on Illumina (Illumina, Inc., San Diego, CA) BeadChip Arrays Sentrix Human-6 (46k probesets). Typically, 0.5-1 µg of RNA combined from three to five independent transduction experiments was used in labeling reactions and hybridization with the arrays according to the manufacturer's instructions. Data was analyzed using the BeadStudio v4 Gene Expression Module (Illumina, Inc.) and Genespring (Agilent, Amstelveen, The Netherlands).

**Flow cytometry analysis**

Cell counting and viability measurements were done using an Accuri C6 (Beckton Dickinson, Breda, the Netherlands) or MACSquant (Miltenyi Biotech) flowcytometer and data was analyzed using WinList 3D (Topsham, USA) or FlowJo (Tree Star, Oregon, USA) software.

**Western blotting**

For western blotting, cells ($1x10^6$/group) were incubated in normoxia or hypoxia for the indicated time. To prevent degradation of HIF proteins during preparation of the lysates, cells were fixed with 1% formaldehyde directly in the culture medium for 10 minutes, after which the formaldehyde was quenched by adding 0,1 volume of 1,25 M glycine for 2 minutes. Cells were subsequently pelleted and washed once with PBS. After centrifugation, the pellet was resuspended in 100 µl Laemmli SDS lysis buffer and boiled

for 15 minutes to reverse the crosslinks and to reduce the sample. 25 µl of lysate was then run on 4-15% polyacrylamide gels (Bio-Rad) and blotted onto PVDF membrane using a Trans-Blot turbo device (Bio-Rad). After blotting, membranes were blocked in 5% skim milk iin PBS for 30 minutes and probed with antibodies against HIF1 (NB100-134, R&D systems), HIF2, (NB100-122, R&D systems) and ARNT (NB100-110, R&D systems), in a 1:1000 dilution overnight at 4°C. Membranes were subsequently washed and incubated with Alexa680 labeled secondary antibodies (1:20.000, 2 hours), washed and scanned on an Odyssey near infrared scanner (Li-Cor Biosciences, Lincoln, NE, USA).

**Animal experiments**

8- to 10-week-old female NSG (NOD.Cg-Prkdcscid Il2rgtm1Wjl/SzJ) were purchased from the Central Animal Facility breeding facility within the UMCG. Mouse experiments were performed in accordance with national and institutional guidelines, and all experiments were approved by the Institutional Animal Care and Use Committee of the University of Groningen. Prior to transplantations, mice were sublethally irradiated with a dose of 1.0 Gy. After irradiation, mice received Neomycin (3.5 g/L in drinking water). Mice were lateral tail vein injected with $1x10^6$ K562 cells, either wild type, HIF1, HIF2 or ARNT knockout (five mice per group). Mice were bled after 24 and 41 days to check human (K562) chimerism in the peripheral blood by flow cytometric analysis using CD99-PE antibodies (BD biosciences). Mice were sacrificed when tumor volumes reached ethical limits or when showing severe signs of illness. After sacrifice, cells were isolated from the bone marrow, spleen, liver and tumor (when present) and human chimerism was determined. Viable tumor cells were isolated by density gradient

centrifugation using Lymphoprep (Alere technologies, Oslo, Norway). 7.25x$10^6$ tumor cells per sample were then extracted as above.

**References**


1. Fellmann C, Hoffmann T, Sridhar V, Hopfgartner B, Muhar M, Roth M, et al. An Optimized microRNA Backbone for Effective Single-Copy RNAi. Cell Rep. 2013;5**:**1704-1713.

2. Schuringa JJ, Chung KY, Morrone G, Moore MA. Constitutive activation of STAT5A promotes human hematopoietic stem cell self-renewal and erythroid differentiation. J Exp Med. 2004;200**:**623-635.

3. Wierenga AT, Vellenga E, Schuringa JJ. Maximal STAT5-induced proliferation and self-renewal at intermediate STAT5 activity levels. Mol Cell Biol. 2008;28**:**6668-6680.

4. Wierenga AT, Vellenga E, Schuringa JJ. Convergence of hypoxia and TGFbeta pathways on cell cycle regulation in human hematopoietic stem/progenitor cells. PLoS One. 2014;9**:**e93494.

5. Chen B, Gilbert LA, Cimini BA, Schnitzbauer J, Zhang W, Li GW, et al. Dynamic imaging of genomic loci in living human cells by an optimized CRISPR/Cas system. Cell. 2013;155**:**1479-1491.

6. Ran FA, Hsu PD, Wright J, Agarwala V, Scott DA, Zhang F. Genome engineering using the CRISPR-Cas9 system. Nat Protoc. 2013;8**:**2281-2308.

7. Li H, Durbin R. Fast and accurate short read alignment with Burrows-Wheeler transform. Bioinformatics. 2009;25**:**1754-1760.

8. Li H, Handsaker B, Wysoker A, Fennell T, Ruan J, Homer N, et al. The Sequence Alignment/Map format and SAMtools. Bioinformatics. 2009;25**:**2078-2079.

9. Kent WJ, Sugnet CW, Furey TS, Roskin KM, Pringle TH, Zahler AM, et al. The human genome browser at UCSC. Genome Res. 2002;12**:**996-1006.

10. McLean CY, Bristor D, Hiller M, Clarke SL, Schaar BT, Lowe CB, et al. GREAT improves functional interpretation of cis-regulatory regions. Nat Biotechnol. 2010;28**:**495-501.

11. Georgiou G, van Heeringen SJ. fluff: exploratory analysis and visualization of high-throughput sequencing data. PeerJ. 2016;4**:**e2209.

12. Afgan E, Baker D, van den Beek M, Blankenberg D, Bouvier D, Cech M, et al. The Galaxy platform for accessible, reproducible and collaborative biomedical analyses: 2016 update. Nucleic Acids Res. 2016;44**:**W3-W10.

13. Cox J, Mann M. MaxQuant enables high peptide identification rates, individualized p.p.b.-range mass accuracies and proteome-wide protein quantification. Nat Biotechnol. 2008;26**:**1367-1372.

14. Tyanova S, Temu T, Sinitcyn P, Carlson A, Hein MY, Geiger T, et al. The Perseus computational platform for comprehensive analysis of (prote)omics data. Nat Methods. 2016;13**:**731-740.

15. Ludwig C, Gunther UL. MetaboLab--advanced NMR data processing and analysis for metabolomics. BMC Bioinformatics. 2011;12**:**366.
